# Exercise-based interventions for postpartum depressive symptoms: a systematic review and network meta-analysis of exercise modality and weekly volume

**DOI:** 10.3389/fpsyt.2026.1921342

**Published:** 2026-09-30

**Authors:** Shiqi Liu, Yanli Tan, Liuhong Zang

**Affiliations:** Institute of Physical Education, Xinjiang Normal University, Urumqi, China

**Keywords:** exercise, network meta-analysis, perinatal mental health, postpartum care, postpartum depressive symptoms

## Abstract

**Objectives:**

To synthesize randomized evidence on exercise-based interventions for postpartum depressive symptom scores and to explore comparative signals across exercise types and pragmatic weekly-volume categories using pairwise and network meta-analysis.

**Methods:**

Eight bibliographic databases were searched from inception and updated through July 23, 2026, with three trial registries searched in the final update, for randomized controlled trials of postpartum exercise interventions reporting Edinburgh Postnatal Depression Scale (EPDS) scores. Pairwise meta-analysis used mean differences (MDs), and network meta-analyses evaluated exercise-type and weekly-volume nodes. The review was registered in PROSPERO (CRD420251275738).

**Results:**

Twenty-four randomized controlled trials involving 1, 792 unique participants (912 intervention and 880 control) were included. Exercise was associated with lower EPDS scores than usual care/non-exercise control (MD = -2.73, 95% confidence interval (CI) -3.57 to -1.88; I^2^ = 95%), representing an average effect across highly heterogeneous studies. Delivery format showed no difference in an omnibus comparison across three reported categories (p = 0.29), and intervention composition also showed no subgroup difference (p = 0.66). Interventions lasting <12 weeks showed a larger pooled effect than those lasting ≥12 weeks (subgroup p = 0.009), but this was exploratory. In the exercise-type network, yoga/mind-body exercise ranked highest (surface under the cumulative ranking curve (SUCRA) = 94.2%) and was estimated to lower EPDS scores versus aerobic exercise (MD = -2.62, 95% CI -5.16 to -0.08); active-vs-active estimates were entirely indirect and network certainty was very low. The 45–90 min/week category ranked highest in the weekly-volume network (SUCRA = 84.6%), but no between-volume contrast was statistically significant.

**Conclusion:**

Exercise may reduce postpartum depressive symptom scores, but mixed prevention-oriented and treatment-oriented samples, substantial heterogeneity, co-interventions, and star-shaped networks preclude identifying an optimal exercise type or dose. Exercise may be considered as an adjunct within individualized postpartum mental-health care, not a substitute for clinical assessment or indicated treatment.

**Systematic review registration:** https://www.crd.york.ac.uk/PROSPERO/view/CRD420251275738, identifier CRD420251275738.

## Introduction

Postpartum depressive symptoms are common and clinically consequential during the first year after childbirth, and clinically diagnosed postpartum depression (PPD) is one important presentation within the broader perinatal mental-health spectrum. In this review, prevention-oriented exercise refers to support offered to women with low or subclinical symptom levels, whereas treatment-oriented exercise refers to symptom-reduction programs enrolling women with elevated screening scores or higher symptom-based eligibility thresholds. An elevated Edinburgh Postnatal Depression Scale (EPDS) score indicates symptom burden or risk but does not by itself establish a clinical diagnosis. Symptoms may coincide with physical recovery, infant-care demands, sleep disruption, and changing family roles (1). Population estimates indicate that approximately 10-20% of women experience perinatal or postpartum depressive symptoms, with considerable variation by screening method, timing, and population characteristics (2). The burden appears higher in many low- and middle-income settings, where social adversity, limited access to care, and weaker health-system infrastructure may compound risk (3). Persistent or untreated symptoms can undermine maternal functioning, mother-infant interaction, breastfeeding confidence, and child cognitive and socioemotional development (4). Symptoms may also emerge or continue beyond the earliest postnatal screening period, which makes interventions that remain feasible across the first postpartum year clinically important (5).

Although evidence-based care for clinically significant postpartum depression includes psychological therapies and pharmacotherapy, treatment access and acceptability remain uneven across postpartum populations. Contemporary clinical guidance emphasizes screening, assessment of severity, suicide-risk evaluation when indicated, and individualized treatment planning rather than a single uniform pathway (6). In practice, however, many women do not receive timely care because of stigma, limited specialist availability, cost, competing childcare demands, and uncertainty about whether symptoms warrant formal treatment (7). Medication decisions can also be complicated by preferences around breastfeeding, concerns about infant exposure, and the perceived trade-off between maternal symptom relief and medication safety (8). These issues do not reduce the importance of medication or psychotherapy for women who need them, but they strengthen the rationale for acceptable, low-cost, and scalable non-pharmacological options that can complement clinical care, support self-management, and provide an early step in stepped-care models (9). Reviews of perinatal depression management have similarly emphasized that postpartum care should be flexible, patient-centered, and responsive to the practical constraints faced by women after birth (10).

Physical activity and structured exercise are plausible postpartum mental-health interventions because they can be adapted to different functional levels, delivered individually or in groups, and integrated into community or home routines. In the general adult population, exercise has demonstrated antidepressant effects across randomized trials and meta-analytic evidence (11). Postpartum-specific evidence suggests that physical activity can reduce depressive and anxiety symptom scores, although effects vary by intervention type, baseline symptom severity, delivery mode, supervision, and adherence (12). Earlier reviews of perinatal and postpartum exercise interventions have supported the overall promise of exercise while highlighting heterogeneity in trial designs and intervention prescriptions (13, 14). This heterogeneity has practical consequences: walking, cycling, aerobic exercise, Pilates, and yoga-informed programs differ in intensity, social context, accessibility, and the relative contribution of movement, breathing, relaxation, and psychological components. Public-health guidance generally encourages postpartum women without contraindications to progress toward regular moderate-intensity activity, but clinical decisions for women with depressive symptoms often require more specific information about feasible modalities and weekly exercise volumes (15).

Despite increasing evidence, several questions remain about how comparative exercise evidence should be interpreted in postpartum mental-health care. Conventional pairwise meta-analysis can estimate whether exercise is associated with lower symptom scores than usual care or other non-exercise control conditions, but it is less able to compare several active exercise types when head-to-head trials are sparse. Recent network meta-analyses have begun to address this problem, but uncertainty remains about how Yoga/mind-body exercise, Walking/cycling, and Aerobic exercise compare when newer trials and Chinese-language evidence are considered (16, 17). An overview of systematic reviews confirmed the relevance of physical-activity interventions while noting that secondary-evidence syntheses are limited in their ability to generate detailed exercise-prescription guidance (18). The present review focuses on randomized trials reporting EPDS outcomes, incorporates evidence from English- and Chinese-language databases, and evaluates exercise type and pragmatic weekly-volume categories within a single comparative framework. It synthesizes a mixed prevention-oriented and treatment-oriented evidence base; it does not assume that all participants had clinically diagnosed PPD. Rather than proposing a definitive exercise prescription, the review aims to identify cautious, clinically interpretable signals that may inform individualized postpartum mental-health support and future head-to-head trials.

## Methods

This systematic review followed the Preferred Reporting Items for Systematic Reviews and Meta-Analyses (PRISMA) 2020 statement and the PRISMA extension for network meta-analysis where applicable (19, 20), and was registered in PROSPERO (CRD420251275738). Because this study synthesized published aggregate data and did not involve direct contact with human participants, institutional review board approval and informed consent were not required.

### Eligibility criteria

Eligibility criteria were structured according to PICOS. Participants were women aged 18 years or older assessed within 12 months after childbirth. Interventions were exercise-based programs initiated during the postpartum period. Comparators were usual care or non-exercise control conditions, including routine follow-up, health education, social support, psychotherapy, or other non-exercise approaches. The primary outcome was the Edinburgh Postnatal Depression Scale (EPDS), a 10-item self-report measure used to screen, rather than diagnose, postpartum depressive symptoms, measured at baseline and after the intervention (21). Requiring EPDS outcomes maintained a common measurement scale across trials and permitted pooling as mean differences without cross-scale standardization. Eligible study designs were randomized controlled trials.

Studies were excluded if exercise began during pregnancy, EPDS data were unavailable, the intervention was not exercise-based, or outcome data could not be extracted. Samples defined by serious physical illness or a major mental disorder, such as bipolar disorder or psychotic depression, were also excluded when these conditions were central characteristics of the recruited population. This restriction improved population comparability but may limit generalizability to women with complex psychiatric or medical histories.

### Information sources and search strategy

Two reviewers independently searched PubMed, Embase, Web of Science Core Collection, the Cochrane Library, CINAHL via EBSCOhost, China National Knowledge Infrastructure (CNKI), Wanfang Data, and the VIP Database. The original searches were conducted through December 15, 2025 and were updated and broadened on July 23, 2026; ClinicalTrials.gov, the WHO International Clinical Trials Registry Platform (ICTRP), and the Chinese Clinical Trial Registry (ChiCTR) were also searched in the final update. The updated strategies used three principal concept blocks—postpartum status, depression or depressive symptoms, and exercise or physical activity—and did not require EPDS or randomized-trial terminology as mandatory search blocks. The updated searches were not restricted to abstract fields. No language filter was applied during database searching, although eligible reports were restricted to English- or Chinese-language publications. No publication-status or document-type restrictions were applied at the search stage. Reference lists of relevant reviews and eligible articles were screened manually, yielding two additional records through citation searching. Duplicate citations were removed electronically and then verified manually. Database- and registry-specific strategies, search dates, fields, and record counts are provided in Supplementary Material 1.

### Study selection

After deduplication, two reviewers independently screened titles and abstracts and then assessed full texts for eligibility. Disagreements were resolved through discussion, and a third reviewer adjudicated unresolved discrepancies. Screening decisions and exclusion reasons were documented in a PRISMA flow diagram.

### Data extraction and risk-of-bias assessment

Two reviewers independently extracted study characteristics, participant information, intervention details, comparator descriptions, and outcome data; disagreements during data extraction and risk-of-bias assessment were resolved through discussion, with unresolved discrepancies adjudicated by a third reviewer (Liuhong Zang). Extracted variables included country, age, EPDS eligibility threshold, group-specific baseline EPDS mean ± SD, completed follow-up sample size at the selected post-intervention EPDS assessment, intervention format, intervention components, postpartum start time, intervention duration, weekly exercise dose, and post-intervention EPDS values. When required outcome data were missing, unclear, or incompletely reported, the corresponding author was contacted up to three times over a three-week period. When a standard error (SE) was reported instead of an SD, it was converted using SD = SE × √n. No other missing EPDS outcome values were statistically derived or imputed. When multiple eligible post-baseline assessments were reported, the assessment corresponding most closely to the end of the active intervention period was selected for the primary analysis; later assessments after cessation of the active intervention were treated as follow-up measurements and were not used as the primary post-intervention endpoint. Baseline EPDS values were used to describe variation in symptom level across trials, not as standardized clinical diagnoses. When both change scores and post-intervention scores were available, post-intervention EPDS scores were used for the primary analysis to maintain consistency across trials. Baseline imbalances were not statistically adjusted in the study-level mean differences; instead, baseline EPDS values were extracted and considered when interpreting heterogeneity, transitivity, and clinical comparability. Because the two active intervention arms in Liu et al. (22) were assigned to the same yoga/mind-body exercise analytical node, they were combined before analysis using standard formulas for combining groups: means were weighted by sample size, and the combined standard deviation incorporated both within-arm variance and between-arm differences in means. The shared control group was included only once. The combined active group comprised 57 participants (baseline EPDS 8.43 ± 4.44; post-intervention EPDS 6.70 ± 3.87), and the control group comprised 33 participants (baseline EPDS 8.98 ± 3.97; post-intervention EPDS 7.76 ± 4.10). Risk of bias was assessed independently with Cochrane RoB 2 for the effect of assignment to the randomized intervention on the post-intervention EPDS outcome. Domains were D1 randomization process, D2 deviations from intended interventions, D3 missing outcome data, D4 outcome measurement, and D5 selection of the reported result; overall judgments were Low risk, Some concerns, or High risk (23). For D5, the availability of prespecified outcome information and the potential for selection among multiple eligible outcome measurements or analyses were considered when judging bias in selection of the reported result. The full trial report was the primary source, supplemented by trial registrations, protocols, or other prespecified outcome information where available.

### Intervention grouping

For subgroup analyses, interventions were classified according to three contextual features: delivery format (Individual/home-based exercise, Group-based exercise, or Format unclear/not reported), intervention composition (Exercise-only or Exercise plus co-intervention), and duration (<12 weeks or ≥12 weeks). The Format unclear/not reported category indicates insufficient reporting in the original study and is not a distinct clinically meaningful delivery strategy. The delivery-format subgroup-difference test was specified as an omnibus comparison across all three reported categories. These subgroup comparisons were intended to explore contextual sources of heterogeneity rather than to establish optimal program characteristics.

For the exercise-type network meta-analysis, the node taxonomy was defined pragmatically from the prescribed physical exercise and interventions were classified by their dominant movement component into Walking/cycling, Aerobic exercise, Yoga/mind-body exercise, and the common comparator node Usual care/non-exercise control. Walking/cycling included walking, pram walking or pushing, and cycling/pedal-based programs. Aerobic exercise included structured aerobic, gymnastic, or cardiorespiratory programs and mixed exercise programs when aerobic/cardiorespiratory activity was the predominant prescribed physical component. Yoga/mind-body exercise included yoga, laughter yoga, Pilates, mindfulness-yoga, and related programs when structured postures or movement integrated with breathing, relaxation, or mindfulness formed a core physical component. When programs contained multiple exercise or non-exercise elements, node assignment was based on the principal prescribed physical activity described in the intervention protocol rather than on adjunctive psychological, educational, digital, social-support, or nutrition/hygiene components. This pragmatic classification organized heterogeneous interventions but did not imply equivalence of all active ingredients within a node; accordingly, network estimates should not be interpreted as pure exercise-type effects.

For the weekly-volume network, weekly volume was derived from the prescribed dose information available in each trial. Fixed frequency and duration were multiplied directly; midpoint values were used for reported ranges, minimum values when only a minimum dose was specified, and reported weekly means were retained. If a reported dose range crossed categories, the resulting representative value determined node assignment. After dose extraction, studies were grouped pragmatically into 45-90, 91-150, and 151–360 min/week to organize the observed distribution into analyzable network nodes; these intervals were evidence-grouping categories rather than clinical prescription thresholds. Usual care/non-exercise control constituted the common comparator node. Because classification was based on prescribed rather than achieved exercise volume, it did not incorporate adherence or attendance.

### Statistical analysis

Pairwise meta-analyses were performed in Review Manager 5.4 using mean differences (MDs) with 95% confidence intervals (CIs), because all included studies reported EPDS scores on the same scale. Heterogeneity was assessed using the I^2^ statistic and Cochran’s Q test (24). Fixed-effect models were used when I^2^ ≤ 50% and the Q-test p value was > 0.10; random-effects models were used when I^2^ > 50% or p ≤ 0.10. Random-effects models were used when heterogeneity criteria were met because a common true effect was not assumed across clinically and methodologically heterogeneous trials. For random-effects analyses, between-study variance (τ^2^) was estimated using the DerSimonian-Laird method. A 95% prediction interval was calculated for the overall random-effects estimate to describe the expected range of true effects in a new comparable study.

Network meta-analyses were performed in Stata 17.0 using the network suite, with multivariate meta-analytic estimation implemented through mvmeta, under a frequentist random-effects consistency framework and with a common heterogeneity variance (τ^2^) estimated by restricted maximum likelihood (REML) within each network. Network plots displayed the geometry of the available evidence, with node size proportional to sample size and edge thickness proportional to the number of direct comparisons. Transitivity was assessed descriptively by comparing EPDS eligibility and baseline symptom levels, postpartum timing, intervention duration, supervision, co-interventions, and control-condition type across nodes; no formal between-node tests were performed. Distributions are reported in **Supplementary Tables S5, S6**. The common comparator node is labeled Usual care/non-exercise control and includes routine follow-up or other eligible non-exercise conditions. Comparator conditions were additionally classified as passive/usual-care (no trial-specific structured non-exercise intervention beyond routine care) or active (a trial-specific structured non-exercise intervention); both were retained within the primary Control node to preserve network connectivity without assuming clinical equivalence. Both evidence networks were star-shaped, centered on this common comparator, and lacked closed loops. Active-vs-active estimates were therefore entirely indirect, and incoherence could not be empirically evaluated. Treatment rankings were summarized using the surface under the cumulative ranking curve (SUCRA) only as exploratory ranking signals, not as evidence of a best intervention or optimal dose. Sensitivity analyses included the existing leave-one-study-out approach, an active-versus-passive comparator subgroup analysis, and pairwise, exercise-type network, and weekly-volume network analyses restricted to passive/usual-care-controlled trials; detailed results are provided in **Supplementary Tables S1–S4**. Publication bias was assessed with funnel plots and Egger’s regression test.

Certainty of the main network estimates was assessed using a structured CINeMA-informed framework covering within-study bias, reporting bias, indirectness, imprecision, heterogeneity, and incoherence (25). For imprecision and heterogeneity judgments, a 4-point EPDS difference was used as an evidence-informed clinically important threshold, defining a symmetric equivalence range of -4 to +4 points. This choice was informed by a 4-point reliable-change criterion derived for the EPDS in a postnatal sample and a 4-point MCID for improvement reported in a pregnancy sample (26, 27); because these thresholds were developed primarily for within-person change, the 4-point value was treated as an evidence-informed decision threshold rather than a universally established between-group MID. Indirectness judgments were informed by the distributions of potential effect modifiers in **Supplementary Tables S5, S6**. Because both networks lacked closed loops, incoherence could not be empirically evaluated; consistent with the CINeMA default rule when the design-by-treatment interaction test is not estimable, this was treated as a major concern in the certainty tables. Overall confidence ratings were summarized as high, moderate, low, or very low and are reported in **Supplementary Tables S7, S8**.

## Results

### Study selection

The updated search identified 6, 206 records in total: 6, 204 from bibliographic databases and trial registries and two through citation searching. The broadened July 23, 2026 update identified no additional eligible trials. After removal of 2, 787 duplicates, 3, 419 records were screened, of which 2, 759 were excluded. Of 660 reports sought for retrieval, eight were not retrieved; 652 reports were assessed for eligibility and 628 were excluded, leaving 24 randomized controlled trials for quantitative synthesis (**Figure 1**).

**Figure 1:**
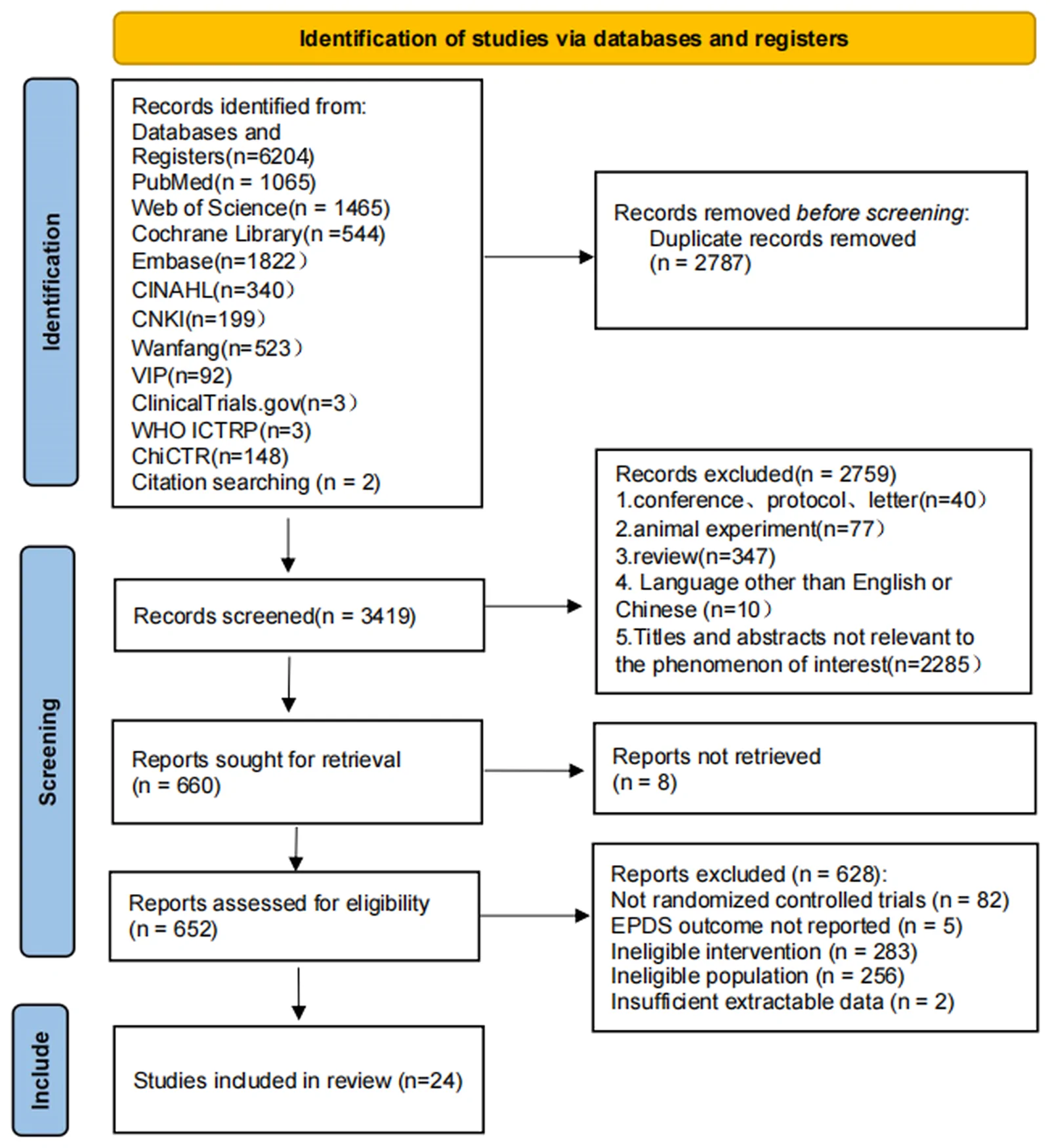
PRISMA flow diagram of study identification, screening, eligibility assessment, and inclusion. PRISMA, Preferred Reporting Items for Systematic Reviews and Meta-Analyses; RCT, randomized controlled trial.

### Study characteristics

The 24 included trials contributed 24 independent study-level comparisons involving 1, 792 unique participants (912 intervention and 880 control) from eight countries or regions. For Liu et al. (22), the two active arms were combined before analysis and the shared control was included once. Interventions were initiated between the first postpartum month and 12 months after childbirth and lasted 4–24 weeks. Delivery format, supervision, and adjunctive non-exercise components varied across studies, as summarized in **Table 1**. Baseline EPDS criteria and scores also varied: some trials were prevention-oriented or early-support studies enrolling women with low or subclinical symptom scores, whereas others were treatment-oriented symptom-reduction studies using higher EPDS eligibility thresholds. Because EPDS is a screening scale, these thresholds do not establish a uniform population with clinically diagnosed PPD. Thirteen studies were conducted in middle-income settings (China, Turkey, or India), whereas none were conducted in low-income countries.

**Table 1:** Characteristics of the included studies.

Exercise type	Study	Country	Age (E/C)	EPDS eligibility criterion	Baseline EPDS, mean ± SD (E/C)	Completed follow-up, n (E/C)	Exercise/adjunct	Comparator	Format/supervision	Postpartum start and duration	Reported exercise dose	Calculated weekly volume (min/week)	Weekly-volume node	Post-intervention EPDS (E)	Post-intervention EPDS (C)
Walking/cycling	Armstrong and Edwards (2003) (28)	Australia	Range: 21–30	EPDS ≥12	17.40 ± 4.65/18.40 ± 4.77	10/10	Pram walking; social support	Non-intervention control; usual routine/social activities with brief telephone support at week 6	Group-based, supervised	Started at 6 weeks–12 months postpartum and lasted 12 weeks	3 times/week, 30–40 min/session	105 (midpoint)	91–150 min/week	4.60 ± 3.34	14.70 ± 7.66
Walking/cycling	Armstrong and Edwards (2004) (29)	Australia	Majority aged 30 years	EPDS ≥12	17.25 ± 4.00/17.17 ± 4.45	9/10	Pram walking; none reported	Non-structured social-support sessions (playgroup-like; no therapeutic intent)	Group-based, supervised	Started at 6 weeks–12 months postpartum and lasted 12 weeks	3 times/week, 40 min/session	120	91–150 min/week	6.33 ± 3.67	13.33 ± 7.66
Walking/cycling	Daley et al. (2008) (30)	United Kingdom	Most were aged 29–31 years	EPDS >12	17.7 ± 5.2/19.2 ± 4.7	16/15	Pram pushing; none reported	Usual care; prescribed medication and counselling/behavioral treatments permitted	Individual/home-based, unsupervised	Started at less than 12 months postpartum and lasted 12 weeks	At least 5 times/week, 30 min/session	150 (minimum)	91–150 min/week	13.1 ± 5.2	14.3 ± 5.4
Walking/cycling	Keller et al. (2014) (31)	United Kingdom	28.3	No EPDS threshold reported	8.21 ± 5.22/8.69 ± 4.71	39/54	Walking; social support	Attention control: monthly non-PA health newsletters plus weekly telephone contact on postpartum/newborn concerns	Group-based, supervised	Started at 6 weeks–6 months postpartum and lasted 24 weeks	150 min/week	150	91–150 min/week	7.05 ± 5.36	7.80 ± 5.05
Walking/cycling	Ren et al. (2019) (32)	China	27.83/28.14	EPDS >9	12.05 ± 1.17/11.26 ± 1.40	19/19	Pedal bicycle; none reported	Usual daily activities; no aerobic training	Format unclear/not reported; supervised	Started at 6 weeks postpartum and lasted 12 weeks	3 times/week, 40 min/session	120	91–150 min/week	9.94 ± 2.32	11.42 ± 2.03
Walking/cycling	Robichaud (2008) (33)	United States	31.1/30.4	No EPDS threshold reported	19.76 ± 4.64/18.87 ± 3.22	25/23	Walking; none reported	Wait-list control; maintain typical activity levels/no aerobic exercise for 6 weeks	Individual/home-based, unsupervised	Started at 6 weeks–12 months postpartum and lasted 6 weeks	3 times/week, 30 min/session	90	45–90 min/week	18.08 ± 3.28	18.39 ± 3.68
Yoga/mind-body exercise	Chen (2025) (34)	China	32.17/32.51	No EPDS threshold reported	14.05 ± 1.24/14.12 ± 1.31	41/41	Yoga; mobile platform	Mobile network platform plus routine postpartum care	Individual/home-based, supervised	Started at 6 weeks postpartum and lasted 4 weeks	3 times/week, 20 min/session	60	45–90 min/week	7.09 ± 1.08	11.12 ± 1.10
Yoga/mind-body exercise	Gao et al. (2021) (35)	China	27.92/28.63	No EPDS threshold reported	5.92 ± 4.53/6.73 ± 4.52	19/12	Yoga; none reported	No rehabilitation intervention; standard postpartum education only	Group-based, supervised	Started at 6–10 weeks postpartum and lasted 12 weeks	3 times/week, 60 min/session	180	151–360 min/week	2.93 ± 2.33	6.18 ± 3.46
Yoga/mind-body exercise	Koca et al. (2025) (36)	Turkey	29.5/29.95	EPDS ≥13	14.600 ± 2.701/14.750 ± 2.677	60/44	Laughter yoga; none reported	No intervention; regular activities	Group-based, supervised	Started at 0–1 month postpartum and lasted 6 weeks	Once/week, 45 min/session	45	45–90 min/week	5.08 ± 3.31	14.07 ± 3.12
Yoga/mind-body exercise	Li and Gao (2019) (37)	China	26.21/26.71	EPDS ≥10	17.69 ± 5.11/17.24 ± 4.39	50/50	Yoga; counseling	Routine perinatal care plus group and individualized psychological counseling	Format unclear/not reported; supervised	Lasted 8 weeks	2 times/week, 60 min/session	120	91–150 min/week	9.68 ± 2.14	11.58 ± 2.31
Yoga/mind-body exercise	Liu et al. (2025) (22)	China	30.48/30.45	No EPDS threshold reported	8.43 ± 4.44/8.98 ± 3.97	57/33	Mindfulness and yoga; VR enhancement in one of the two pooled active arms	Blank/no-intervention control	Group-based, supervised	Lasted 8 weeks	3 times/week, 60 min/session	180	151–360 min/week	6.70 ± 3.87	7.76 ± 4.10
Yoga/mind-body exercise	Wang (2024) (38)	China	29.03/28.56	EPDS ≥13	20.11 ± 2.83/20.25 ± 2.68	50/50	Mindfulness yoga; CBT	Medical–nursing collaborative cognitive-behavioral intervention	Group-based, supervised	Started at 1 month postpartum and lasted 12 weeks	2 times/week, 60 min/session	120	91–150 min/week	13.26 ± 0.94	14.43 ± 1.39
Aerobic exercise	Da Costa et al. (2009) (39)	Canada	34.3/32.7	EPDS ≥10	13.6 ± 3.6/13.6 ± 3.9	46/42	Aerobic/strength/flexibility; none reported	Usual care	Individual/home-based, unsupervised	Started at 4–38 weeks postpartum and lasted 12 weeks	Reported mean: 165 min/week	165 (reported mean)	151–360 min/week	9.26 ± 4.8	9.6 ± 5.42
Aerobic exercise	Daley et al. (2015) (40)	United Kingdom	31.7/29.3	EPDS ≥13	17.3 ± 3.0/17.5 ± 3.7	43/42	Aerobic; none reported	Usual care only; antidepressants and/or psychological treatment allowed as clinically provided	Individual/home-based, unsupervised	Started within 6 months after childbirth and lasted 24 weeks	3–5 times/week, cumulating 30 min/session	120 (midpoint)	91–150 min/week	12.51 ± 5.46	14.67 ± 4.86
Aerobic exercise	Forsyth et al. (2017) (41)	United Kingdom	25/27	EPDS ≥12	17.6 ± 4.0/15.9 ± 2.9	11/11	Gymnastic; none reported	Usual healthcare program	Group-based, supervised	Started at 6 weeks postpartum and lasted 12 weeks	Reported mean: 61.6 min/week	61.6 (reported mean)	45–90 min/week	11.8 ± 6.1	12.7 ± 4.2
Aerobic exercise	Haruna et al. (2013) (42)	Japan	33.8/33.7	No EPDS threshold reported	4.1 ± 4.0/5.9 ± 3.8	48/47	Aerobic; none reported	Wait-list/no exercise program during the study; exercise program offered after outcome measurement	Group-based, supervised	Started at 3 months postpartum and lasted 4 weeks	4 times/week, 90 min/session	360	151–360 min/week	3.6 ± 4.20	4.10 ± 3.40
Aerobic exercise	Mei (2024) (43)	China	37.85/37.23	EPDS >9	11.32 ± 1.05/12.87 ± 1.34	63/62	Aerobic; none reported	Family supportive education	Format unclear/not reported; supervised	Lasted 12 weeks	2–3 times/week, 30–40 min/session	87.5 (midpoint)	45–90 min/week	9.01 ± 1.01	10.98 ± 1.02
Aerobic exercise	Norman et al. (2010) (44)	Australia	29.3/30.1	No EPDS threshold reported	8.00 ± 6.16/6.75 ± 5.44	62/73	Aerobic; parent education	Education-only: weekly written postnatal/parenting educational material	Group-based, supervised	Started at 6–10 weeks and lasted 8 weeks	Once/week, 60 min/session	60	45–90 min/week	5.47 ± 5.11	6.75 ± 5.51
Aerobic exercise	Teychenne et al. (2021) (45)	Australia	33.6/33.0	EPDS ≥10	12.1 ± 3.8/12.6 ± 3.9	32/30	Aerobic; social support	Usual routine	Individual/home-based, unsupervised	Started at 3–9 months postpartum and lasted 12 weeks	Reported mean: 57.4 min/week	57.4 (reported mean)	45–90 min/week	6.0 ± 4.3	7.4 ± 3.6
Aerobic exercise	Thiruppathi et al. (2014) (46)	India	26.3/25.1	No EPDS threshold reported	7.95 ± 0.75/7.76 ± 0.62	20/21	Aerobic; none reported	Health Care Education Only: the same weekly written educational material as the intervention group	Group-based, supervised	Started at 4 weeks postpartum and lasted 8 weeks	Once/week, 45 min/session	45	45–90 min/week	4.5 ± 0.6	7.72 ± 0.46
Aerobic exercise	Wang and Zhao (2025) (47)	China	28.64/28.52	No EPDS threshold reported	11.85 ± 1.45/11.92 ± 1.48	42/42	Aerobic; CBT	Cognitive-behavioral intervention	Group-based, supervised	NA	3 times/week, 35 min/session	105	91–150 min/week	9.26 ± 1.18	10.79 ± 1.24
Aerobic exercise	Yang and Chen (2018) (48)	China	31.89/32.45	No EPDS threshold reported	9.11 ± 5.54/8.45 ± 4.68	60/62	Aerobic gymnastics; none reported	Regular postpartum care, including postnatal exercise handouts/booklets	Individual/home-based, unsupervised	Started at 6 weeks postpartum and lasted 12 weeks	At least 3 times/week, 15 min/session	45 (minimum)	45–90 min/week	7.60 ± 4.71	7.18 ± 4.54
Yoga/mind-body exercise	Özcan and Eryilmaz (2024) (49)	Turkey	25.8/25.1	EPDS ≥12	14.75 ± 2.88/16.09 ± 3.55	56/56	Pilates; nutrition/hygiene	Routine postpartum care (standard nursing care with breastfeeding education; no study home visits during the intervention period)	Individual/home-based, unsupervised	Started at 4 weeks postpartum and lasted 8 weeks	At least 2–3 times/week, 20–30 min/session	62.5 (midpoint)	45–90 min/week	5.59 ± 3.74	15.45 ± 2.21
Aerobic exercise	Özkan et al. (2020) (50)	Turkey	28.90 ± 4.83	EPDS ≥13	16.41 ± 1.61/15.74 ± 2.35	34/31	ACOG-based activity; none reported	Standard postpartum care practices at the family health center	Individual/home-based, unsupervised	Started at 1 month postpartum and lasted 4 weeks	At least 5 days/week, at least 30 min/day	150 (minimum)	91–150 min/week	7.29 ± 1.67	12.54 ± 2.65

E, experimental group; C, control group; EPDS, Edinburgh Postnatal Depression Scale; PP, postpartum; SD, standard deviation; NA, not available. Age and EPDS values are as reported; sample size is E/C. “None reported” indicates no adjunctive non-exercise component was reported; “Format unclear/not reported” indicates that the original report did not permit a clear individual/home-based or group-based classification.

Exercise content was organized into the analytical nodes Walking/cycling, Aerobic exercise, Yoga/mind-body exercise, and Usual care/non-exercise control. Active programs were delivered alone or with co-interventions. All included studies assessed depressive symptoms using the EPDS. Because several yoga/mind-body exercise and aerobic exercise programs included psychological, educational, digital, social-support, or nutrition/hygiene components, network estimates should not be interpreted as pure exercise-only effects.

### Risk of bias

Overall RoB 2 judgments were Some concerns for 20 studies and High risk for four studies (29, 33, 48, 50); none were Low risk. All 24 trials had Some concerns in D4 because EPDS was participant-reported and participants were generally aware of assignment (**Figures 2**, **3**).

**Figure 2:**
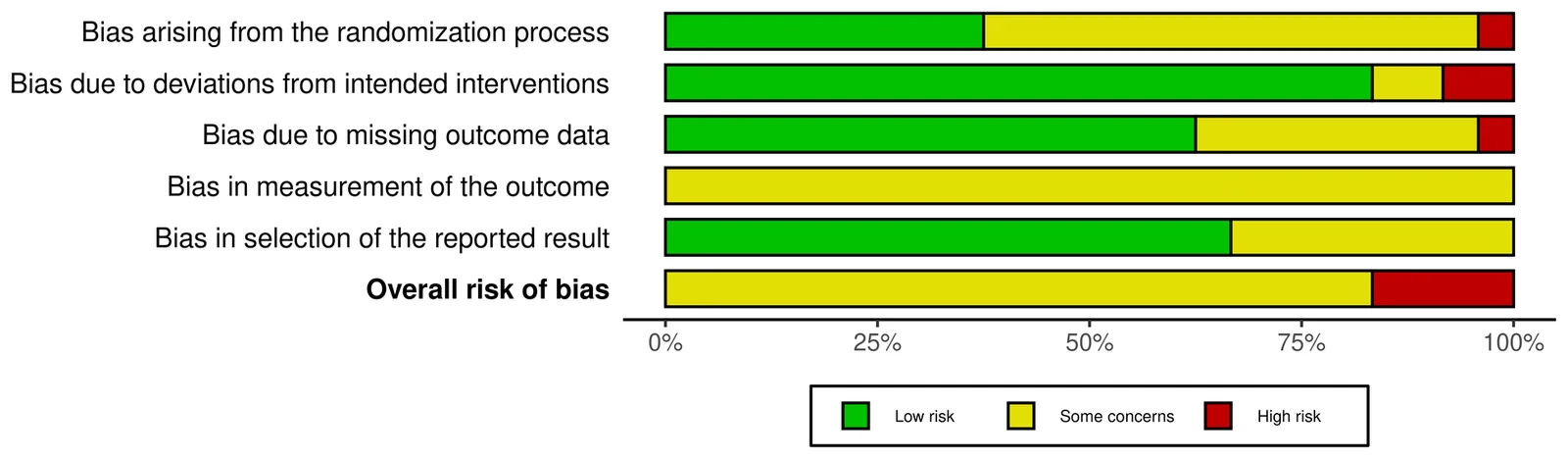
RoB 2 risk-of-bias graph. Percentages of low risk, some concerns, and high risk judgments across D1-D5 and overall.

**Figure 3:**
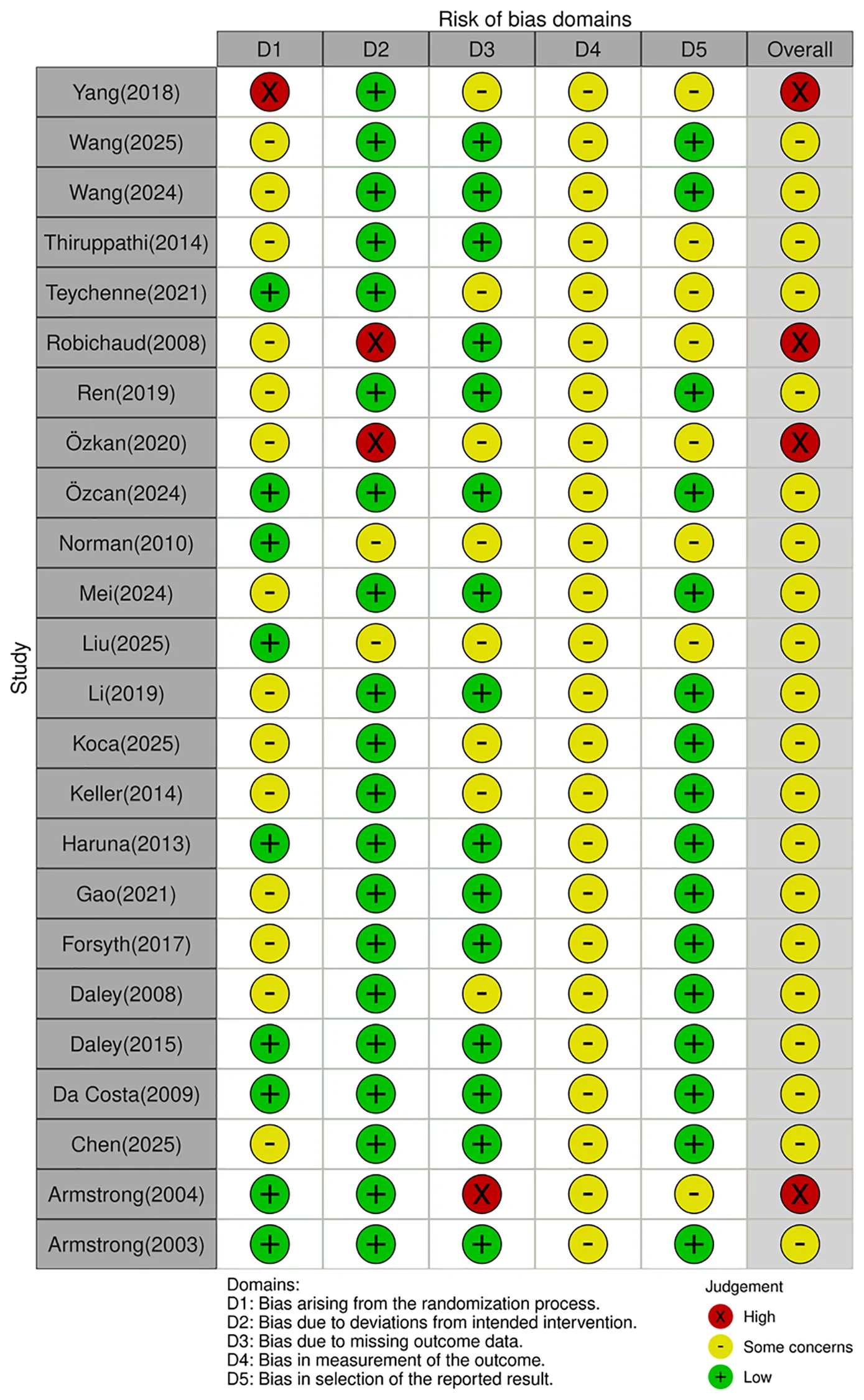
RoB 2 risk-of-bias summary. D1-D5 represent randomization, deviations from intended interventions, missing outcome data, outcome measurement, and selection of the reported result. Green, yellow, and red indicate Low risk, some concerns, and high risk, respectively.

### Pairwise meta-analysis

The pooled pairwise meta-analysis indicated that exercise was associated with lower postpartum depressive symptom scores than usual care/non-exercise control (MD = -2.73, 95% CI -3.57 to -1.88; I^2^ = 95%) (**Figure 4**). The 95% prediction interval was -6.50 to 1.05, indicating that the true effect in a new comparable study could range from a larger reduction in EPDS scores to little or no benefit. This MD is an average effect across highly heterogeneous populations and interventions and should not be interpreted as a common effect expected in all postpartum populations.

**Figure 4:**
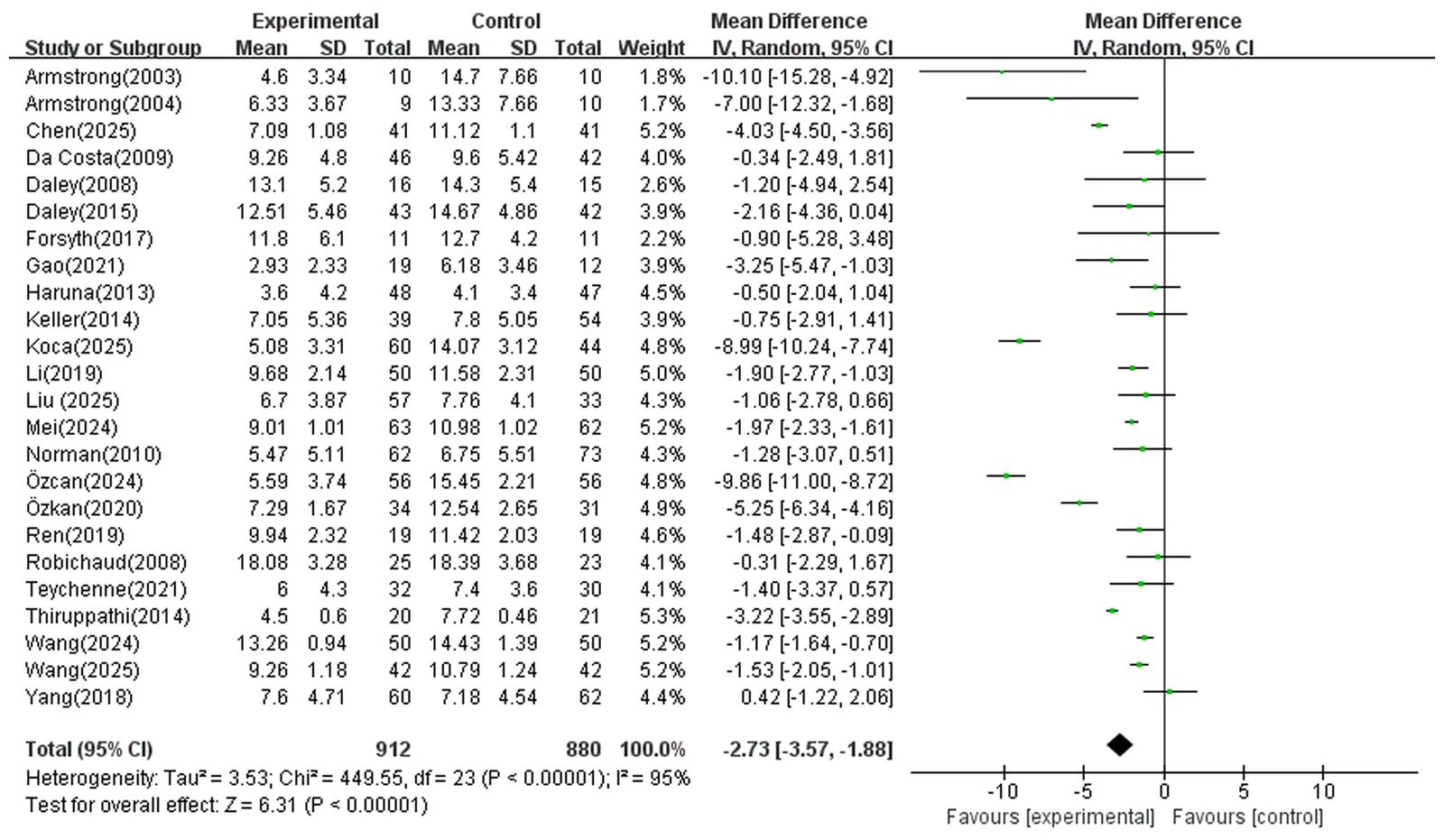
Forest plot of the overall effect of postpartum exercise on EPDS scores. Negative mean differences favor exercise over usual care or other non-exercise control conditions. EPDS, Edinburgh Postnatal Depression Scale; MD, mean difference; CI, confidence interval; SD, standard deviation; IV, inverse variance.

In the delivery-format subgroup, pooled estimates favored exercise over usual care/non-exercise control for Individual/home-based exercise (MD = -2.80, 95% CI -4.89 to -0.71; I^2^ = 95%), Group-based exercise (MD = -2.83, 95% CI -4.06 to -1.61; I^2^ = 94%), and Format unclear/not reported (MD = -1.93, 95% CI -2.25 to -1.61; I^2^ = 0%). The omnibus subgroup-difference test across all three categories showed no evidence of a difference (p = 0.29) (**Figure 5**; it was not a pairwise comparison of Group-based exercise with Individual/home-based exercise).

**Figure 5:**
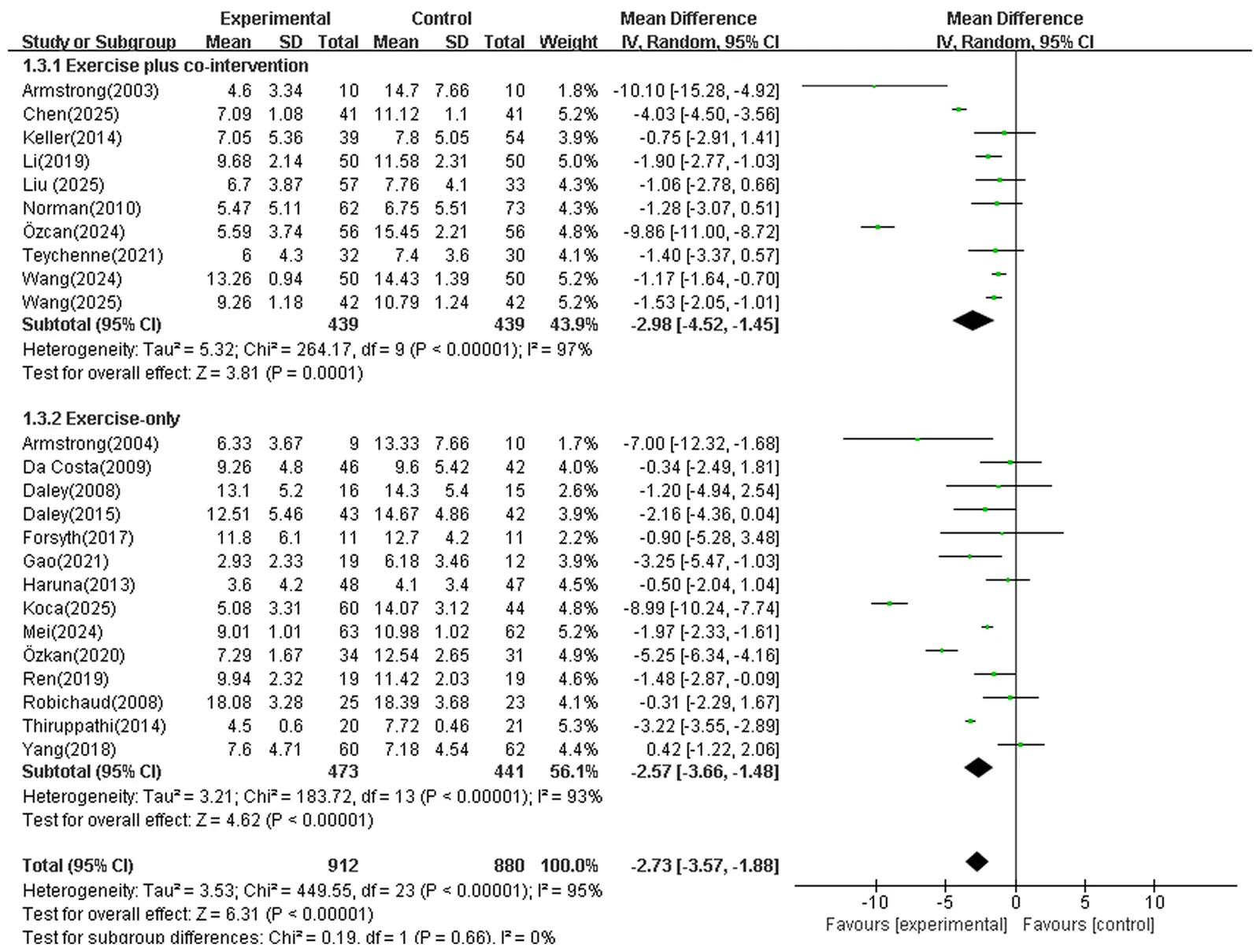
Forest plot stratified by reported intervention delivery format. Delivery format was categorized as Individual/home-based exercise, group-based exercise, or format unclear/not reported. The Format unclear/not reported category reflects incomplete intervention reporting rather than a distinct delivery strategy. The subgroup-difference test is an omnibus comparison across all three categories. Negative mean differences favor exercise over usual care/non-exercise control. EPDS, Edinburgh Postnatal Depression Scale; MD, mean difference; CI, confidence interval; SD, standard deviation; IV, inverse variance.

In the intervention-composition subgroup, pooled estimates favored Exercise plus co-intervention (MD = -2.98, 95% CI -4.52 to -1.45; I^2^ = 97%) and Exercise-only (MD = -2.57, 95% CI -3.66 to -1.48; I^2^ = 93%) over usual care/non-exercise control. There was no statistically significant subgroup difference (p = 0.66) (**Figure 6**).

**Figure 6:**
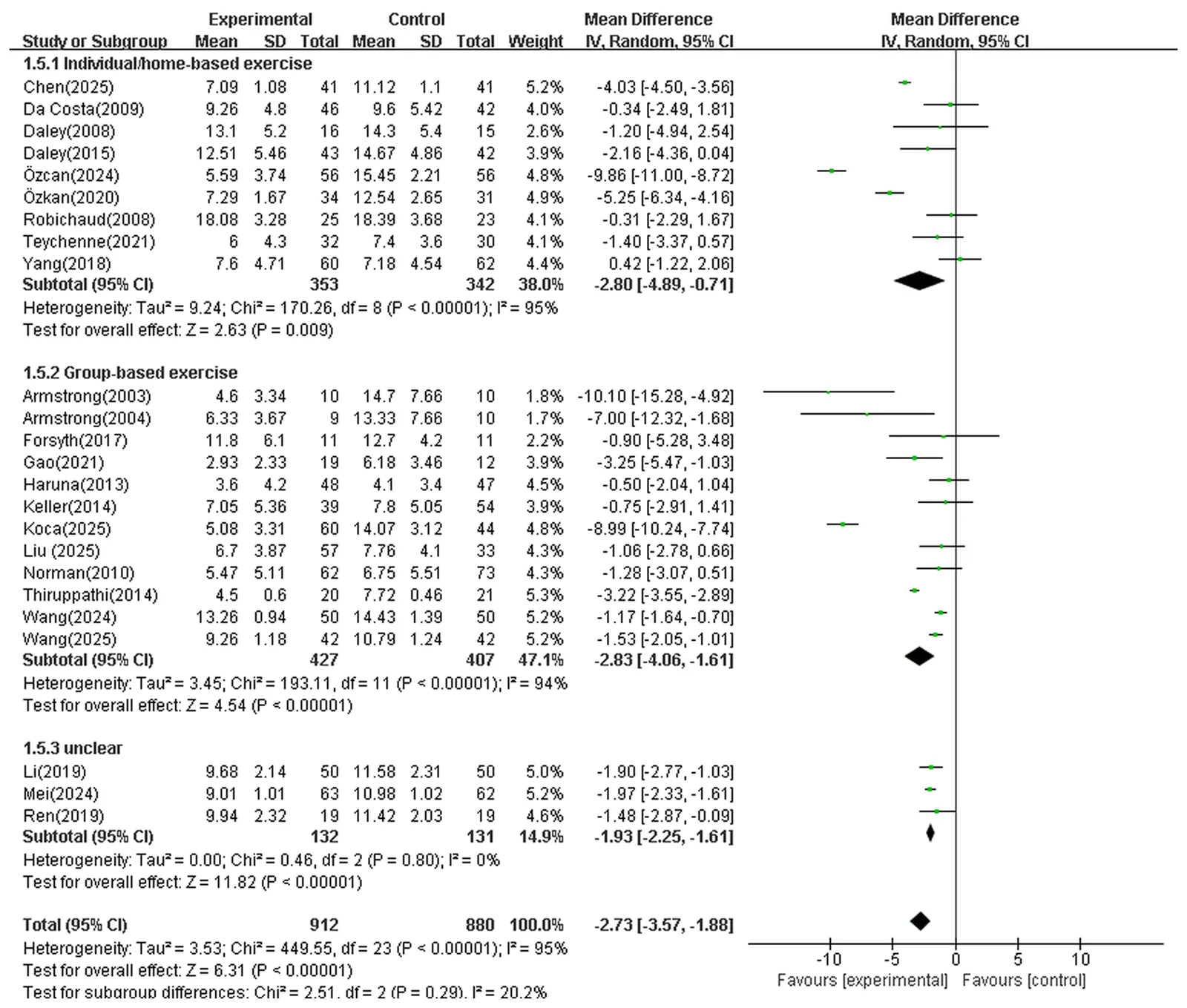
Forest plot stratified by intervention composition. Negative mean differences favor exercise over usual care/non-exercise control. Exercise plus co-intervention refers to exercise delivered with adjunctive non-exercise components such as education, psychological counseling, mindfulness, CBT, social support, VR, digital support, or nutrition/hygiene support. EPDS, Edinburgh Postnatal Depression Scale; MD, mean difference; CI, confidence interval; SD, standard deviation; IV, inverse variance.

Intervention duration was the only contextual factor with a statistically significant subgroup-difference signal (p = 0.009) (**Figure 7**). Programs lasting <12 weeks had a pooled MD of -3.72 (95% CI -5.18 to -2.26; I^2^ = 97%), whereas programs lasting ≥12 weeks had a pooled MD of -1.57 (95% CI -2.24 to -0.89; I^2^ = 62%). Because heterogeneity remained very high in the <12-week subgroup and the comparison was exploratory, this pattern should not be interpreted as evidence of an optimal duration.

**Figure 7:**
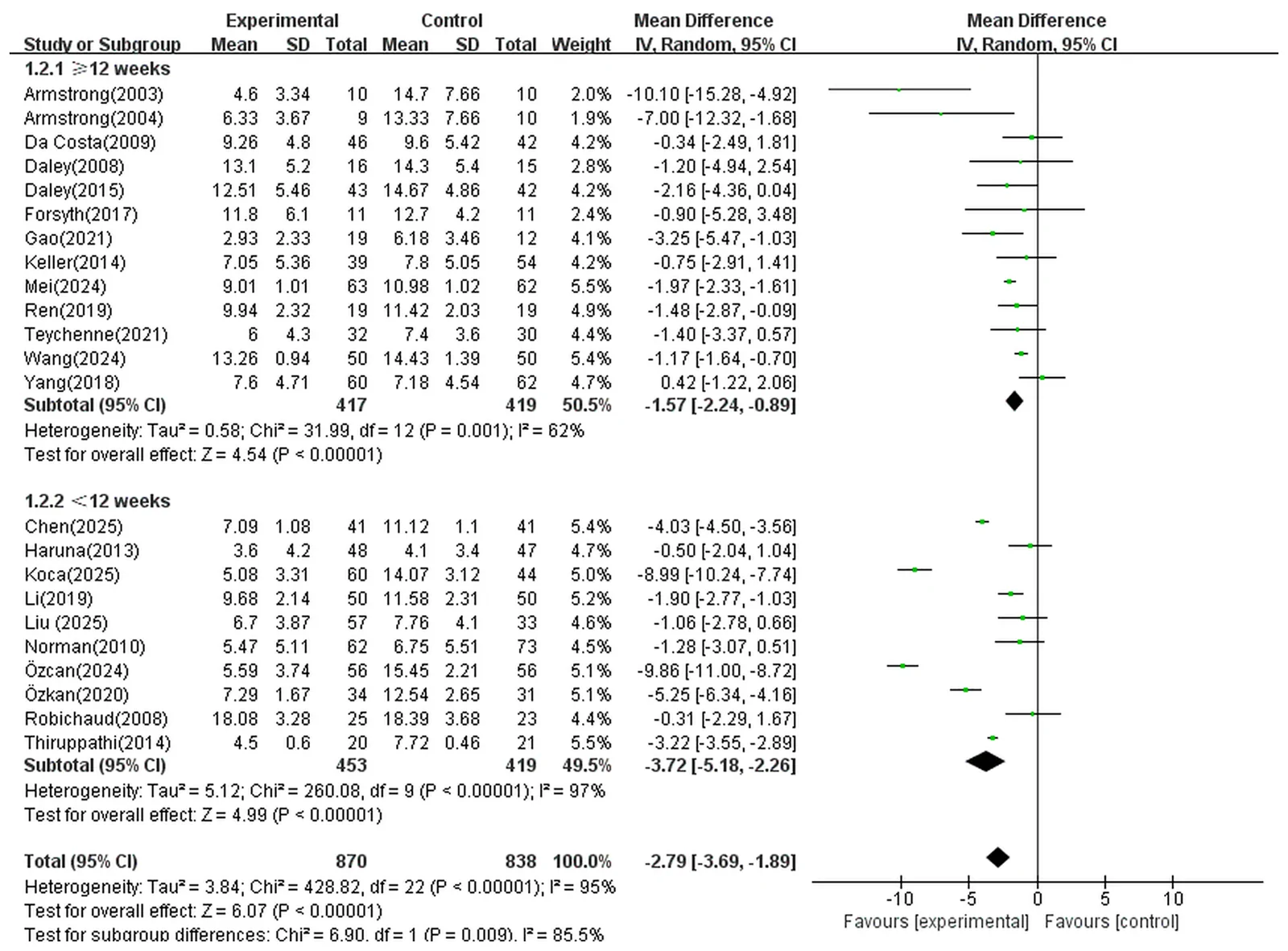
Forest plot stratified by intervention duration (<12 weeks vs ≥12 weeks). Negative mean differences favor exercise over usual care/non-exercise control. EPDS, Edinburgh Postnatal Depression Scale; MD, mean difference; CI, confidence interval. The subgroup-difference p value is 0.009. The finding is exploratory and should not be interpreted as an optimal-duration recommendation because residual heterogeneity was high. One trial with unreported intervention duration was excluded from this subgroup analysis but was retained in the other eligible analyses.

### Network meta-analysis

In the exercise-type network, all 24 studies contributed data across Walking/cycling (6 direct comparisons), Aerobic exercise (11), Yoga/mind-body exercise (7), and Usual care/non-exercise control (**Figure 8**). SUCRA values were 94.2% for Yoga/mind-body exercise, 62.2% for Walking/cycling, 42.3% for Aerobic exercise, and 1.3% for Usual care/non-exercise control. The common heterogeneity variance was τ^2^ = 6.59. The estimated comparison of Yoga/mind-body exercise with Aerobic exercise favored Yoga/mind-body exercise (MD = -2.62, 95% CI -5.16 to -0.08) (**Table 2**). This is an exploratory, entirely indirect active-vs-active comparison: the star-shaped network lacked closed loops, incoherence could not be empirically evaluated, and several active programs included co-interventions. The comparison and SUCRA ranking should be interpreted in light of the very-low-certainty evidence in the CINeMA-informed assessment (**Supplementary Table S7**).

**Figure 8:**
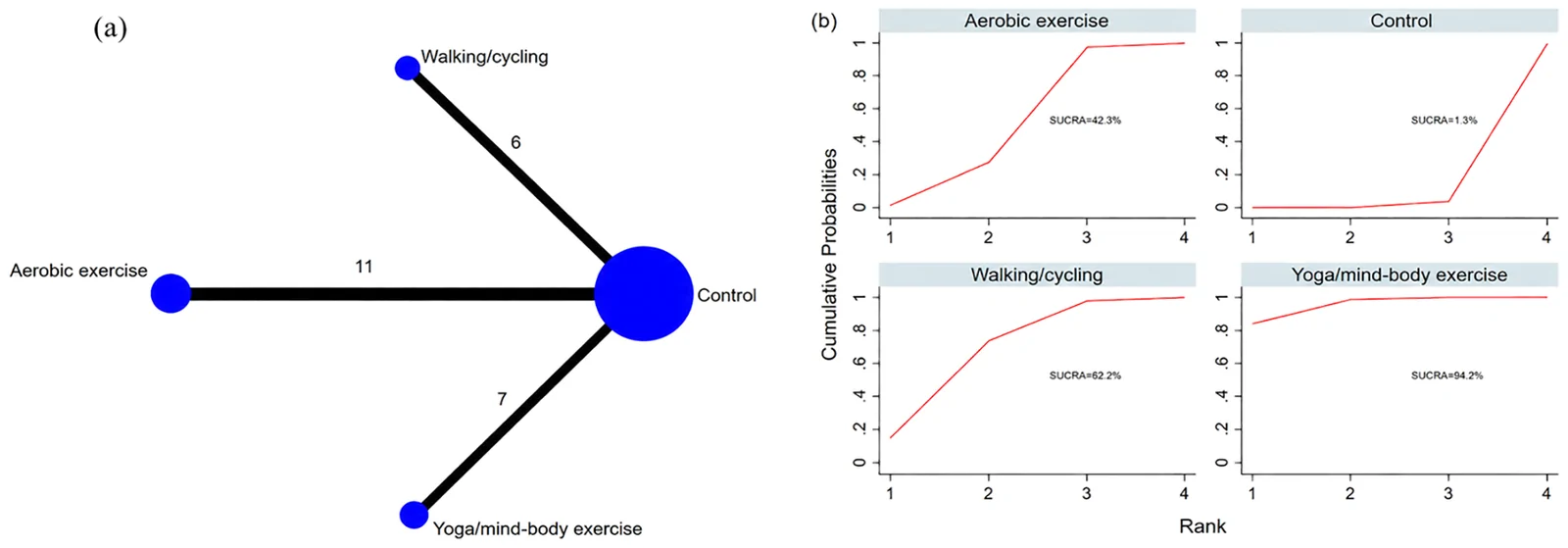
Exercise-type network plot and SUCRA ranking curves. Panel **(A)** shows network geometry and Panel **(B)** shows SUCRA ranking curves for Walking/cycling, Aerobic exercise, Yoga/mind-body exercise, and Usual care/non-exercise control. Node size is proportional to the number of participants, and edge thickness is proportional to the number of direct comparisons. Rankings are exploratory and should be interpreted in light of very-low-certainty evidence; the star-shaped network is centered on a common control, lacks closed loops, active-vs-active estimates are entirely indirect, and incoherence cannot be empirically evaluated.

**Table 2:** League table for the network meta-analysis of exercise type.

Exercise type	Walking/cycling	Aerobic exercise	Yoga/mind-body exercise	Control
Walking/cycling	0			
Aerobic exercise	-0.94 (-3.89, 2.01)	0		
Yoga/mind-body exercise	1.68 (-1.47, 4.83)	2.62 (0.08, 5.16)*	0	
Control	-2.65 (-5.12, -0.19)*	-1.71 (-3.32, -0.10)*	-4.33 (-6.30, -2.37)*	0

Values are mean differences (95% confidence intervals) in post-intervention EPDS scores for Walking/cycling, Aerobic exercise, Yoga/mind-body exercise, and Usual care/non-exercise control. Negative values favor the column intervention over the row intervention. EPDS, Edinburgh Postnatal Depression Scale. *95% confidence interval excludes 0.

In the weekly-volume network, all 24 studies contributed data across 45–90 min/week, 91–150 min/week, 151–360 min/week, and Usual care/non-exercise control (**Figure 9**). SUCRA values were 84.6%, 71.9%, 36.9%, and 6.5%, respectively. The common heterogeneity variance was τ^2^ = 7.52. None of the contrasts among the three active weekly-volume nodes was statistically significant. The SUCRA ordering is therefore an exploratory ranking signal that should be interpreted in light of very-low-certainty evidence and does not establish an optimal weekly exercise dose (**Table 3**; **Supplementary Table S8**).

**Figure 9:**
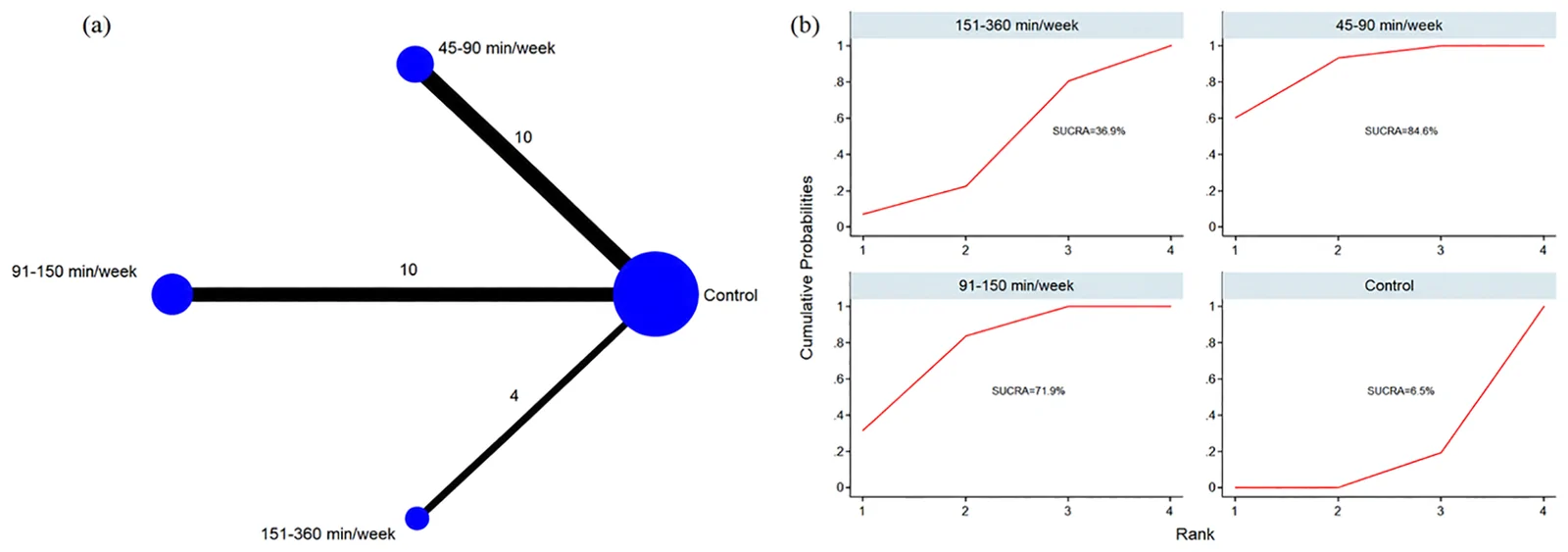
Weekly-volume network plot and SUCRA ranking curves. **(A)** shows network geometry and **(B)** shows SUCRA ranking curves for 45–90 min/week, 91–150 min/week, 151–360 min/week, and Usual care/non-exercise control. Node size is proportional to the number of participants, and edge thickness is proportional to the number of direct comparisons. Rankings are exploratory and should be interpreted in light of very-low-certainty evidence; between-volume contrasts were not statistically significant. The star-shaped network lacks closed loops, active-vs-active estimates are entirely indirect, and incoherence cannot be empirically evaluated; no optimal weekly dose can be inferred.

**Table 3:** League table for the network meta-analysis of weekly exercise volume.

Weekly volume	45–90 min/week	91–150 min/week	151–360 min/week	Control
45–90 min/week	0			
91–150 min/week	-0.52 (-3.10, 2.07)	0		
151–360 min/week	-2.04 (-5.40, 1.33)	-1.52 (-4.93, 1.89)	0	
Control	-3.30 (-5.09, -1.52)*	-2.79 (-4.65, -0.92)*	-1.27 (-4.12, 1.59)	0

Values are mean differences (95% confidence intervals) in post-intervention EPDS scores. Negative values favor the column intervention over the row intervention. EPDS, Edinburgh Postnatal Depression Scale. *95% confidence interval excludes 0.

Descriptive assessment showed some imbalance in potential effect modifiers across nodes. Yoga/mind-body trials more often included co-interventions and supervised delivery, while weekly-volume nodes differed more clearly in baseline EPDS, EPDS-based eligibility, postpartum timing, and comparator type; for example, active comparators occurred in 40.0%, 50.0%, and 0.0% of the 45-90, 91-150, and 151–360 min/week nodes, respectively. Transitivity was therefore considered plausible only with caution, particularly for the weekly-volume network (**Supplementary Tables S5, S6**).

Certainty was rated very low for all six main comparisons in each network (**Supplementary Tables S7, S8**). Within-study bias raised some concerns because none of the included trials was overall low risk in RoB 2, whereas reporting bias was rated as undetected, with limited power at the comparison level. Indirectness raised some concerns for exercise-type active-vs-active comparisons and major concerns for weekly-volume active-vs-active comparisons because of the effect-modifier imbalances described above. Imprecision and heterogeneity were judged relative to the -4 to +4 EPDS equivalence range using the corresponding 95% CIs and prediction intervals. Because neither network contained a closed loop, incoherence could not be empirically evaluated; under the CINeMA default rule, this non-estimability was treated as a major concern and contributed to the very-low overall confidence.

### Sensitivity analysis

Leave-one-out analyses indicated that omission of any single study did not materially change the direction or statistical significance of the overall pooled effect (**Table 4**). The pooled MD remained close to the main estimate in all iterations; however, heterogeneity remained substantial in every iteration (I^2^ = 92%-95%). The analysis therefore supports robustness of the pooled effect direction and statistical significance to single-study omission, but not consistency of effect magnitudes across studies.

**Table 4:** Leave-one-out sensitivity analysis for the overall pairwise meta-analysis.

Excluded study	Pooled MD	95% CI	P value	I^2^
Overall analysis (all studies)	-2.73	-3.57, -1.88	p<0.00001	95%
Armstrong (28)	-2.59	-3.44, -1.74	p<0.00001	95%
Armstrong (29)	-2.65	-3.51, -1.80	p<0.00001	95%
Chen (34)	-2.66	-3.55, -1.76	p<0.00001	95%
Da Costa (39)	-2.82	-3.69, -1.96	p<0.00001	95%
Daley (30)	-2.77	-3.63, -1.91	p<0.00001	95%
Daley (40)	-2.75	-3.62, -1.88	p<0.00001	95%
Forsyth (41)	-2.77	-3.62, -1.91	p<0.00001	95%
Gao (35)	-2.71	-3.57, -1.84	p<0.00001	95%
Haruna (42)	-2.83	-3.70, -1.96	p<0.00001	95%
Keller (31)	-2.81	-3.67, -1.94	p<0.00001	95%
Koca (36)	-2.41	-3.19, -1.62	p<0.00001	94%
Li (37)	-2.77	-3.66, -1.89	p<0.00001	95%
Liu (22)	-2.80	-3.67, -1.93	p<0.00001	95%
Mei (43)	-2.77	-3.73, -1.82	p<0.00001	95%
Norman (44)	-2.79	-3.66, -1.92	p<0.00001	95%
Ren (32)	-2.79	-3.66, -1.91	p<0.00001	95%
Robichaud (33)	-2.83	-3.69, -1.97	p<0.00001	95%
Teychenne (45)	-2.78	-3.65, -1.92	p<0.00001	95%
Thiruppathi (46)	-2.71	-3.68, -1.73	p<0.00001	95%
Wang (38)	-2.81	-3.70, -1.92	p<0.00001	95%
Wang (47)	-2.79	-3.69, -1.89	p<0.00001	95%
Yang (48)	-2.87	-3.73, -2.01	p<0.00001	95%
Özcan (49)	-2.35	-3.08, -1.62	p<0.00001	92%
Özkan (50)	-2.60	-3.46, -1.74	p<0.00001	95%

MD, mean difference; CI, confidence interval; I^2^, inconsistency statistic. Negative MD values favor exercise over usual care or other non-exercise control conditions. The overall analysis includes all eligible comparisons, and each subsequent row shows the pooled estimate after excluding the indicated study.

Comparator-type subgroup analysis yielded pooled MDs of -3.04 (95% CI -5.09 to -0.99) for passive/usual-care comparators and -2.21 (95% CI -3.01 to -1.40) for active comparators, with no subgroup difference (p = 0.459). Restricting the pairwise analysis to the 15 passive/usual-care-controlled trials preserved the direction of effect (MD = -3.04, 95% CI -5.09 to -0.99), although heterogeneity remained very high (I^2^ = 95%). In the passive-only exercise-type network, Yoga/mind-body exercise remained highest ranked and favored Aerobic exercise (MD = -4.40, 95% CI -8.23 to -0.57). In the passive-only weekly-volume network, 45–90 and 91–150 min/week were nearly tied (SUCRA = 77.9% and 77.7%, respectively; MD = 0.03, 95% CI -4.38 to 4.44). Full results are provided in **Supplementary Tables S1–S4**.

### Publication bias

The pairwise funnel plot was broadly symmetrical, and Egger’s regression did not indicate significant small-study effects (p = 0.81; exact bias-term p = 0.8052) (**Table 5**). Comparison-adjusted funnel plots for the exercise-type and weekly-volume networks were interpreted cautiously; Egger’s regression tests did not indicate statistically significant small-study effects (p = 0.93 and p = 0.44, respectively), while visual asymmetry may suggest small-study effects and the limited number of studies per comparison constrained interpretation (**Supplementary Figures S1, S2** in **Supplementary Material 1**).

**Table 5:** Egger regression test for the overall pairwise meta-analysis.

Term	Coefficient	SE	t	P>|t|	95% CI lower	95% CI upper
Slope	-2.5606	0.5807	-4.4095	0.0002	-3.7649	-1.3563
Bias	-0.3531	1.4151	-0.2496	0.8052	-3.2879	2.5816

SE, standard error; CI, confidence interval. The bias term represents the Egger regression intercept used to assess small-study effects. A non-significant bias term indicates no statistical evidence of funnel plot asymmetry.

### Consolidated summary of findings

To consolidate the main findings, **Table 6** summarizes the overall meta-analytic result, the subgroup analyses, and the key network rankings.

**Table 6:** Summary of key contextual findings across the pooled and network analyses.

Domain	Comparison/category	Main result	Interpretation
Overall meta-analysis	Exercise vs Usual care/non-exercise control	MD = -2.73 (95% CI -3.57 to -1.88) I^2^ = 95%	Average effect across highly heterogeneous studies; not a common effect expected in all postpartum populations.
Delivery-format subgroup	Individual/home-based exercise Group-based exercise Format unclear/not reported	-2.80 (-4.89 to -0.71), I^2^ = 95% -2.83 (-4.06 to -1.61), I^2^ = 94% -1.93 (-2.25 to -1.61), I^2^ = 0% Omnibus p = 0.29	No evidence of differences across the three reported categories; Format unclear/not reported reflects incomplete reporting and is not a clinical delivery strategy.
Intervention composition	Exercise plus co-intervention Exercise-only	-2.98 (-4.52 to -1.45), I^2^ = 97% -2.57 (-3.66 to -1.48), I^2^ = 93% Subgroup p = 0.66	No statistically significant subgroup difference.
Duration subgroup	<12 weeks ≥12 weeks	-3.72 (-5.18 to -2.26), I^2^ = 97% -1.57 (-2.24 to -0.89), I^2^ = 62% Subgroup p = 0.009	Exploratory subgroup signal; not an optimal-duration recommendation.
Exercise-type NMA	Yoga/mind-body exercise Yoga/mind-body exercise vs Aerobic exercise	SUCRA = 94.2% MD = -2.62 (95% CI -5.16 to -0.08)	Exploratory, entirely indirect active-vs-active comparison; star-shaped network without closed loops and co-intervention concerns.
Weekly-volume NMA	45–90 min/week Active volume-node contrasts	SUCRA = 84.6% No statistically significant between-volume contrasts	Exploratory ranking signal; no optimal weekly dose can be inferred.

MD, mean difference; CI, confidence interval; EPDS, Edinburgh Postnatal Depression Scale; SUCRA, surface under the cumulative ranking curve. Pooled estimates are average effects across heterogeneous studies. SUCRA values are exploratory ranking signals and should be interpreted together with the corresponding forest plots, league tables, and network plots.

## Discussion

### Principal findings

This review found that exercise was associated with lower postpartum depressive symptom scores than usual care/non-exercise control (MD = -2.73, 95% CI -3.57 to -1.88), but the pooled evidence was highly heterogeneous (I^2^ = 95%). The estimate therefore represents an average across substantially different populations and interventions, not a common effect expected in every postpartum population. The average between-group difference of 2.73 EPDS points was smaller than the 4-point evidence-informed threshold used for the certainty assessment, while the 95% prediction interval (-6.50 to 1.05) extended from a potentially larger benefit to little or no effect. Because the 4-point threshold is derived mainly from within-person change and is not a universally established between-group minimal important difference, the clinical relevance of the pooled MD depends on baseline symptom severity, population characteristics, and the context in which change is interpreted. Delivery format did not differ in an omnibus comparison across Individual/home-based exercise, Group-based exercise, and Format unclear/not reported (p = 0.29), and intervention composition also showed no subgroup difference (p = 0.66). The duration comparison favored programs lasting <12 weeks (p = 0.009), but substantial residual heterogeneity limits causal interpretation. In the exercise-type network, yoga/mind-body exercise ranked highest and showed an estimated advantage over aerobic exercise, whereas 45–90 min/week ranked highest in the weekly-volume network (SUCRA = 84.6%) without statistically significant between-volume contrasts. These are exploratory, hypothesis-generating signals, and the CINeMA-informed certainty was very low for all main network comparisons (**Supplementary Tables S7, S8**); neither the league-table contrasts nor SUCRA rankings should therefore be interpreted as establishing superiority or an optimal dose. Comparator type did not significantly modify the pooled effect (p = 0.459), and restricting analyses to passive/usual-care controls preserved the direction of the pairwise effect and the Yoga/mind-body ranking. However, heterogeneity remained high and the 45–90 and 91–150 min/week nodes became nearly indistinguishable, indicating that comparator composition alone did not explain the observed heterogeneity and further weakening any claim of an optimal weekly dose. The transitivity assessment also supports cautious interpretation: exercise-type nodes differed in co-interventions and supervision, and weekly-volume nodes showed clearer imbalance in baseline symptom burden, eligibility, postpartum timing, and comparator composition, so active-vs-active network estimates may partly reflect effect-modifier differences rather than exercise type or volume alone.

The favorable ranking signal for yoga/mind-body exercise should not be interpreted as a specific effect of movement alone. Yoga-informed programs may combine gentle physical postures with breathing regulation, relaxation, and attentional focus, elements that may support stress regulation and mood (51). In the postpartum period, these features may be relevant because women often experience fatigue, sleep disruption, and emotional overload. However, the included yoga/mind-body exercise programs varied in the contribution of active movement, meditation, mindfulness, CBT, counseling, education, VR, social support, digital support, and nutrition/hygiene components. The available evidence therefore cannot separate the contribution of exercise type from the contribution of these adjunctive components.

The subgroup signal for interventions lasting <12 weeks (p = 0.009) should be interpreted as an exploratory study-level association rather than evidence that shorter interventions are intrinsically superior. Residual confounding and ecological bias are plausible because the duration groups also differed across intervention type, baseline symptom severity, co-interventions, comparator conditions, and participant characteristics. Greater feasibility or adherence with shorter programs is one possible explanation, but adherence, attendance, and intervention completion were not systematically evaluated in this review; this explanation therefore remains a hypothesis rather than a finding. Likewise, the higher exploratory ranking of 45–90 min/week does not show that higher volumes are ineffective or that lower volume is optimal. None of the active between-volume contrasts was statistically significant, and the category boundaries were pragmatic evidence-grouping intervals rather than clinical prescription thresholds.

### Implications for perinatal mental health practice

These findings have different implications for prevention-oriented and treatment-oriented postpartum care and should not be translated into a uniform exercise prescription. In prevention-oriented or early-support samples with low or subclinical EPDS scores, exercise may be offered as preference-sensitive support for well-being and symptom management. In treatment-oriented samples with elevated screening scores, exercise may be considered as an adjunct to clinical assessment and indicated care; an EPDS score is not itself a diagnosis. Exercise should not replace diagnostic evaluation, suicide-risk assessment when indicated, psychotherapy, pharmacotherapy, or referral. Community health workers, midwives, nurses, and primary-care providers could offer brief activity guidance and referral pathways, particularly for women with persistent or moderate-to-severe symptoms. Yoga-informed programs may be attractive for some women, but they will not be acceptable, culturally appropriate, affordable, or physically comfortable for everyone. Trauma-informed yoga literature has emphasized that survivors of sexual or interpersonal violence may need choice-based, trauma-sensitive delivery and that standard yoga environments can present barriers if safety and autonomy are not prioritized (52, 53). Walking-based and other low-burden home or community programs therefore remain important alternatives, especially for women who face time, transport, childcare, or financial barriers to participation (54).

### Strengths and limitations

This review has several strengths. It included only randomized trials, searched both English- and Chinese-language databases, used contextual subgroup analyses, and combined pairwise meta-analysis with network meta-analysis to organize evidence on modalities and weekly-volume categories. Independent screening, extraction, and risk-of-bias assessment further strengthened internal consistency. These strengths support a broad synthesis of the available evidence, while the results remain unsuitable for a definitive exercise prescription because of the diversity of interventions and the limited direct comparative evidence.

Several limitations should temper interpretation. First, heterogeneity was substantial in the overall meta-analysis and remained high in several subgroups, so the pooled MD is an average across heterogeneous studies rather than a uniform expected clinical effect. Second, the evidence mixed prevention-oriented or early-support samples with low or subclinical baseline scores and treatment-oriented samples selected using higher EPDS thresholds. Baseline symptom severity and eligibility thresholds varied, EPDS is a screening instrument rather than a diagnostic interview, and the overall result cannot be interpreted as a single treatment effect for clinically diagnosed PPD. Restricting eligibility to EPDS outcomes improved scale comparability but may have excluded otherwise relevant randomized trials that used other validated depression measures. Third, both networks were star-shaped, centered on usual care/non-exercise control, and lacked closed loops. Active-vs-active estimates were therefore entirely indirect, and incoherence could not be empirically evaluated. Fourth, yoga/mind-body exercise and other nodes often contained multicomponent programs, making it difficult to isolate the effect of exercise type. The weekly-volume nodes were based on prescribed dose and pragmatic range, midpoint, and minimum-value rules; classification uncertainty, adherence differences, and within-node heterogeneity may therefore have influenced the volume comparisons. Fifth, some Chinese-language trials provided limited methodological detail, which may have affected risk-of-bias judgments and confidence in pooled estimates. The CINeMA-informed assessment rated certainty as very low for all main network comparisons, reflecting within-study limitations, heterogeneity and indirectness/transitivity concerns, together with the inability to empirically evaluate incoherence in the star-shaped networks. Sixth, although 13 studies were from middle-income countries, no included study was conducted in a low-income country, limiting generalizability to settings where the burden of postpartum depressive symptoms may be greatest. Finally, because populations defined by serious physical illness or major mental disorder were excluded, the findings may be less applicable to women with complex psychiatric or medical histories.

## Conclusion

Across 24 randomized trials involving 1, 792 unique participants, exercise was associated with lower postpartum depressive symptom scores than usual care/non-exercise control (MD = -2.73, 95% CI -3.57 to -1.88; I^2^ = 95%), but this was an average effect across highly heterogeneous studies. Yoga/mind-body exercise ranked highest in the exercise-type network and showed an exploratory estimated advantage over aerobic exercise (MD = -2.62, 95% CI -5.16 to -0.08), while 45–90 min/week ranked highest in the weekly-volume network (SUCRA = 84.6%) without statistically significant differences among active volume categories. The underlying network comparisons were of very low certainty, so these rankings and active-vs-active estimates remain exploratory. Because the evidence mixed prevention-oriented and treatment-oriented samples, included multicomponent interventions, and came from star-shaped networks in which active-vs-active comparisons were entirely indirect and incoherence could not be empirically evaluated, it does not identify an optimal exercise type, duration, or weekly dose. Exercise may be considered as an individualized adjunct within postpartum mental-health care and should not substitute for clinical assessment or indicated treatment.

## Data Availability

The original contributions presented in the study are included in the article/**Supplementary Material**. Further inquiries can be directed to the corresponding author.
