# Supplementary material for: Exercise-based interventions for postpartum depressive symptoms: a systematic review and network meta-analysis of exercise modality and weekly volume
Source: Front Psychiatry. 2026 Sep 30;17:1921342. doi: 10.3389/fpsyt.2026.1921342 (PMC13664296; doi:10.3389/fpsyt.2026.1921342)
Supplement: Supplementary file 1 [file SupplementaryFile1.docx]

# **Supplementary Material 1**

## 1. Database-Specific Search Strategies

Search dates: The original database searches were conducted through December 15, 2025, and were updated and broadened on July 23, 2026. The final updated strategies combined three principal concepts: postpartum status, depression or depressive symptoms, and exercise or physical activity. EPDS and randomized-trial terminology were not used as mandatory search blocks in the updated strategies. Database-specific syntax, field tags, and controlled vocabulary were adapted to each database interface.

## PubMed(n = 1,065)

## (

## "Postpartum Period"[Mesh]

## OR postpartum[Title/Abstract]

## OR "post-partum"[Title/Abstract]

## OR postnatal[Title/Abstract]

## OR "post-natal"[Title/Abstract]

## OR puerper*[Title/Abstract]

## OR "after childbirth"[Title/Abstract]

## OR "after birth"[Title/Abstract]

## )

## AND

## (

## "Depression"[Mesh]

## OR "Depressive Disorder"[Mesh]

## OR "Depression, Postpartum"[Mesh]

## OR depress*[Title/Abstract]

## OR "postpartum depression"[Title/Abstract]

## OR "post-partum depression"[Title/Abstract]

## OR "postnatal depression"[Title/Abstract]

## OR "post-natal depression"[Title/Abstract]

## OR "depressive symptoms"[Title/Abstract]

## )

## AND

## (

## "Exercise"[Mesh]

## OR "Exercise Therapy"[Mesh]

## OR "Motor Activity"[Mesh]

## OR exercis*[Title/Abstract]

## OR "physical activity"[Title/Abstract]

## OR "physical activities"[Title/Abstract]

## OR "exercise training"[Title/Abstract]

## OR "exercise intervention"[Title/Abstract]

## OR walking[Title/Abstract]

## OR cycling[Title/Abstract]

## OR bicycl*[Title/Abstract]

## OR aerobic*[Title/Abstract]

## OR yoga[Title/Abstract]

## OR pilates[Title/Abstract]

## OR fitness[Title/Abstract]

## OR sport*[Title/Abstract]

## OR "mind-body"[Title/Abstract]

## OR "mind body"[Title/Abstract]

## )

## Embase(n = 1,822)

(

'postpartum period'/exp

OR postpartum:ti,ab,kw

OR 'post partum':ti,ab,kw

OR postnatal:ti,ab,kw

OR 'post natal':ti,ab,kw

OR puerper*:ti,ab,kw

OR 'after childbirth':ti,ab,kw

OR 'after birth':ti,ab,kw

)

AND

(

'depression'/exp

OR 'postpartum depression'/exp

OR depress*:ti,ab,kw

OR 'postpartum depression':ti,ab,kw

OR 'post partum depression':ti,ab,kw

OR 'postnatal depression':ti,ab,kw

OR 'post natal depression':ti,ab,kw

OR 'depressive symptoms':ti,ab,kw

)

AND

(

'exercise'/exp

OR 'exercise therapy'/exp

OR 'physical activity'/exp

OR exercis*:ti,ab,kw

OR 'physical activity':ti,ab,kw

OR 'physical activities':ti,ab,kw

OR 'exercise training':ti,ab,kw

OR 'exercise intervention':ti,ab,kw

OR walking:ti,ab,kw

OR cycling:ti,ab,kw

OR bicycl*:ti,ab,kw

OR aerobic*:ti,ab,kw

OR yoga:ti,ab,kw

OR pilates:ti,ab,kw

OR fitness:ti,ab,kw

OR sport*:ti,ab,kw

OR 'mind body':ti,ab,kw

)

## Web of Science Core Collection(n = 1,465)

TS=(

postpartum

OR "post-partum"

OR postnatal

OR "post-natal"

OR puerper*

OR "after childbirth"

OR "after birth"

)

AND

TS=(

depress*

OR "postpartum depression"

OR "post-partum depression"

OR "postnatal depression"

OR "post-natal depression"

OR "depressive symptoms"

)

AND

TS=(

exercis*

OR "physical activity"

OR "physical activities"

OR "exercise training"

OR "exercise intervention"

OR walking

OR cycling

OR bicycl*

OR aerobic*

OR yoga

OR pilates

OR fitness

OR sport*

OR "mind-body"

OR "mind body"

)

## Cochrane Library(n = 544)

(

[mh "Postpartum Period"]

OR postpartum:ti,ab,kw

OR "post-partum":ti,ab,kw

OR postnatal:ti,ab,kw

OR "post-natal":ti,ab,kw

OR puerper*:ti,ab,kw

OR "after childbirth":ti,ab,kw

OR "after birth":ti,ab,kw

)

AND

(

[mh Depression]

OR [mh "Depressive Disorder"]

OR [mh "Depression, Postpartum"]

OR depress*:ti,ab,kw

OR "postpartum depression":ti,ab,kw

OR "post-partum depression":ti,ab,kw

OR "postnatal depression":ti,ab,kw

OR "post-natal depression":ti,ab,kw

OR "depressive symptoms":ti,ab,kw

)

AND

(

[mh Exercise]

OR [mh "Exercise Therapy"]

OR [mh "Motor Activity"]

OR exercis*:ti,ab,kw

OR "physical activity":ti,ab,kw

OR "physical activities":ti,ab,kw

OR "exercise training":ti,ab,kw

OR "exercise intervention":ti,ab,kw

OR walking:ti,ab,kw

OR cycling:ti,ab,kw

OR bicycl*:ti,ab,kw

OR aerobic*:ti,ab,kw

OR yoga:ti,ab,kw

OR pilates:ti,ab,kw

OR fitness:ti,ab,kw

OR sport*:ti,ab,kw

OR "mind-body":ti,ab,kw

OR "mind body":ti,ab,kw

)

## CINAHL(n = 340)

(

TI (postpartum OR "post-partum" OR postnatal OR "post-natal" OR puerper* OR "after childbirth" OR "after birth")

OR

AB (postpartum OR "post-partum" OR postnatal OR "post-natal" OR puerper* OR "after childbirth" OR "after birth")

)

AND

(

TI (depress* OR "postpartum depression" OR "post-partum depression" OR "postnatal depression" OR "post-natal depression" OR "depressive symptoms")

OR

AB (depress* OR "postpartum depression" OR "post-partum depression" OR "postnatal depression" OR "post-natal depression" OR "depressive symptoms")

)

AND

(

TI (exercis* OR "physical activity" OR "physical activities" OR "exercise training" OR "exercise intervention" OR walking OR cycling OR bicycl* OR aerobic* OR yoga OR pilates OR fitness OR sport* OR "mind-body" OR "mind body")

OR

AB (exercis* OR "physical activity" OR "physical activities" OR "exercise training" OR "exercise intervention" OR walking OR cycling OR bicycl* OR aerobic* OR yoga OR pilates OR fitness OR sport* OR "mind-body" OR "mind body")

)

## ClinicalTrials.gov(n = 3)

Condition/disease:

postpartum depression OR postnatal depression

Other terms:

postpartum OR postnatal OR puerperium

Intervention/treatment:

exercise OR "physical activity" OR walking OR cycling OR aerobic OR yoga OR pilates

## WHO ICTRP(n = 3)

(postpartum OR postnatal OR puerperium) AND (depression OR depressive) AND (exercise OR "physical activity" OR walking OR cycling OR aerobic OR yoga OR pilates)

**Chinese Clinical Trial Registry (ChiCTR)(n = 148)**
Search field: Registered title
Search term: 产后抑郁
No restrictions were applied for intervention, study design, registration status, or outcome measure.

## CNKI(n = 199)

主题=(产后 + 产褥 + 产妇 + 分娩后) AND 主题=(抑郁 + 产后抑郁 + 抑郁症 + 抑郁症状 + 抑郁情绪) AND 主题=(运动 + 锻炼 + 体育锻炼 + 身体活动 + 体力活动 + 运动干预 + 运动疗法 + 有氧运动 + 步行 + 骑行 + 瑜伽 + 普拉提 + 健身)

## Wanfang Data(n = 523)

主题:(产后 OR 产褥 OR 产妇 OR 分娩后) AND 主题:(抑郁 OR 抑郁症 OR 抑郁症状 OR 抑郁情绪 OR 产后抑郁) AND 主题:(运动 OR 锻炼 OR 体育锻炼 OR 身体活动 OR 体力活动 OR 运动干预 OR 运动疗法 OR 有氧运动 OR 步行 OR 骑行 OR 瑜伽 OR 普拉提 OR 健身)

## VIP Database(n = 92)

M=(产后 OR 产褥 OR 产妇 OR 分娩后) AND M=(抑郁 OR 产后抑郁 OR 抑郁症 OR 抑郁症状 OR 抑郁情绪) AND M=(运动 OR 锻炼 OR 体育锻炼 OR 身体活动 OR 体力活动 OR 运动干预 OR 运动疗法 OR 运动训练 OR 有氧运动 OR 步行 OR 骑行 OR 自行车 OR 瑜伽 OR 普拉提 OR 健身)

## 2. Supplementary Figures

Supplementary Figure S1. Comparison-adjusted funnel plot for small-study effects in the exercise-type network. Note. The comparison-adjusted funnel plot displays study effects after centering by comparison-specific summary effects. Egger's regression test yielded p = 0.93. Visual asymmetry may suggest small-study effects, although interpretation is limited when the number of studies per comparison is small.

Supplementary Figure S2. Comparison-adjusted funnel plot for small-study effects in the weekly-volume network. Note. The comparison-adjusted funnel plot displays study effects after centering by comparison-specific summary effects. Egger's regression test yielded p = 0.44. Visual asymmetry may suggest small-study effects, although interpretation is limited when the number of studies per comparison is small.

## 3. Supplementary Tables

Supplementary Table S1. Sensitivity analysis by comparator type

| **Comparator type** | **Studies (k)** | **Participants (n)** | **Pooled MD** | **95% CI** | **τ²** | **I² (%)** | **Effect p** | **Q** |
| --- | --- | --- | --- | --- | --- | --- | --- | --- |
| **Passive/usual-care** | 15 | 1013 | -3.04 | -5.09, -0.99 | 14.96 | 95.07 | 0.004 | 284.00 |
| **Active** | 9 | 779 | -2.21 | -3.01, -1.40 | 1.15 | 93.38 | <0.001 | 120.76 |

| Test for subgroup difference | | | |
| --- | --- | --- | --- |
| Comparison | χ² | df | p |
| Active vs Passive/usual-care | 0.55 | 1 | 0.46 |

*Note. Passive/usual-care comparators included no-intervention, wait-list, or routine-care conditions without a trial-specific structured non-exercise intervention; active comparators included trial-specific structured non-exercise interventions.*

Supplementary Table S2. Passive/usual-care-only pairwise sensitivity analysis

| **Analysis** | **Studies (k)** | **Participants (n)** | **Pooled MD** | **95% CI** | **τ²** | **I² (%)** | **Q** | **Q p** |
| --- | --- | --- | --- | --- | --- | --- | --- | --- |
| Main analysis (24 studies) | 24 | 1792 | -2.73 | -3.57, -1.88 | 3.53 | 94.88 | 449.55 | <0.001 |
| Passive/usual-care only | 15 | 1013 | -3.04 | -5.09, -0.99 | 14.96 | 95.07 | 284.00 | <0.001 |

Supplementary Table S3. Passive/usual-care-only exercise-type network meta-analysis

| Node-specific estimates and rankings | | | | |
| --- | --- | --- | --- | --- |
| Node | MD vs Control | 95% CI | SUCRA (%) | Rank |
| Walking/cycling | -2.63 | -5.93, 0.68 | 57.45 | 2 |
| Aerobic exercise | -1.50 | -3.85, 0.85 | 39.97 | 3 |
| Yoga/mind-body exercise | -5.90 | -8.92, -2.88 | 97.07 | 1 |
| Control | 0.00 | 0.00, 0.00 | 5.52 | 4 |

| Pairwise network contrasts | | | | |
| --- | --- | --- | --- | --- |
| Contrast | MD (A−B) | 95% CI | SE | p |
| Walking/cycling vs Aerobic exercise | -1.13 | -5.18, 2.93 | 2.07 | 0.59 |
| Walking/cycling vs Yoga/mind-body exercise | 3.27 | -1.20, 7.75 | 2.28 | 0.15 |
| Walking/cycling vs Control | -2.63 | -5.93, 0.68 | 1.68 | 0.12 |
| Aerobic exercise vs Yoga/mind-body exercise | 4.40 | 0.57, 8.23 | 1.95 | 0.02 |
| Aerobic exercise vs Control | -1.50 | -3.85, 0.85 | 1.20 | 0.21 |
| Yoga/mind-body exercise vs Control | -5.90 | -8.92, -2.88 | 1.54 | <0.001 |

*Note. Negative MD values favor treatment A over treatment B because lower EPDS scores indicate fewer depressive symptoms; for node-specific estimates versus Control, negative values favor the active intervention.*

Supplementary Table S4. Passive/usual-care-only weekly-volume network meta-analysis

| Node-specific estimates and rankings | | | | |
| --- | --- | --- | --- | --- |
| Node | MD vs Control | 95% CI | SUCRA (%) | Rank |
| 45–90 min/week | -3.70 | -6.62, -0.78 | 77.93 | 1 |
| 91–150 min/week | -3.73 | -7.03, -0.42 | 77.72 | 2 |
| 151–360 min/week | -1.27 | -4.81, 2.26 | 35.68 | 3 |
| Control | 0.00 | 0.00, 0.00 | 8.68 | 4 |

| Pairwise network contrasts | | | | |
| --- | --- | --- | --- | --- |
| Contrast | MD (A−B) | 95% CI | SE | p |
| 45–90 min/week vs 91–150 min/week | 0.03 | -4.38, 4.44 | 2.25 | 0.99 |
| 45–90 min/week vs 151–360 min/week | -2.42 | -7.01, 2.16 | 2.34 | 0.30 |
| 45–90 min/week vs Control | -3.70 | -6.62, -0.78 | 1.49 | 0.01 |
| 91–150 min/week vs 151–360 min/week | -2.45 | -7.30, 2.39 | 2.47 | 0.32 |
| 91–150 min/week vs Control | -3.73 | -7.03, -0.42 | 1.69 | 0.03 |
| 151–360 min/week vs Control | -1.27 | -4.81, 2.26 | 1.81 | 0.48 |

*Note. Negative MD values favor treatment A over treatment B because lower EPDS scores indicate fewer depressive symptoms; for node-specific estimates versus Control, negative values favor the active intervention.*

Supplementary Table S5. Distribution of potential effect modifiers across exercise-type nodes

| **Potential effect modifier** | **Walking/cycling** | **Aerobic exercise** | **Yoga/mind-body exercise** |
| --- | --- | --- | --- |
| **Study/sample context** | | | |
| Studies, k | 6 | 11 | 7 |
| Completed follow-up participants, n (E+C) | 249 | 924 | 619 |
| **Baseline symptom burden** | | | |
| Baseline EPDS, median (range) | 17.55 (8.45–19.32) | 12.09 (5.00–17.40) | 14.68 (6.33–20.18) |
| EPDS-based eligibility threshold used, n (%) | 4 (66.7%) | 6 (54.5%) | 4 (57.1%) |
| Reported EPDS eligibility threshold(s) | ≥12; >12; >9 | ≥10; ≥13; ≥12; >9 | ≥13; ≥10; ≥12 |
| **Postpartum timing** | | | |
| Intervention start ≤6 weeks postpartum, n (%) | 1 (16.7%) | 4 (36.4%) | 4 (57.1%) |
| Intervention start >6 weeks postpartum, n (%) | 0 (0.0%) | 2 (18.2%) | 0 (0.0%) |
| Variable/broad start window or not reported, n (%) | 5 (83.3%) | 5 (45.5%) | 3 (42.9%) |
| **Intervention duration** | | | |
| Duration, weeks, median (range) | 12.0 (6–24) | 12.0 (4–24) | 8.0 (4–12) |
| Duration not reported, n | 0 | 1 | 0 |
| **Supervision** | | | |
| Supervised, n (%) | 4 (66.7%) | 6 (54.5%) | 6 (85.7%) |
| Unsupervised, n (%) | 2 (33.3%) | 5 (45.5%) | 1 (14.3%) |
| **Co-intervention** | | | |
| Exercise plus co-intervention, n (%) | 2 (33.3%) | 3 (27.3%) | 5 (71.4%) |
| Exercise-only, n (%) | 4 (66.7%) | 8 (72.7%) | 2 (28.6%) |
| **Comparator type** | | | |
| Active comparator, n (%) | 2 (33.3%) | 4 (36.4%) | 3 (42.9%) |
| Passive/usual-care comparator, n (%) | 4 (66.7%) | 7 (63.6%) | 4 (57.1%) |

*Note. Values are descriptive; no formal between-node hypothesis testing was performed. Baseline EPDS is the median (range) of study-level averages of the intervention- and comparator-arm baseline means. “≤6 weeks” and “>6 weeks” indicate studies whose reported start timing lay wholly within that category; broad/overlapping start windows or unreported timing were classified as variable/broad or not reported. Co-intervention refers to adjunctive non-exercise components in the active arm. Comparator type follows the finalized active versus passive/usual-care classification used in the comparator sensitivity analyses. Percentages use the number of studies within each node as the denominator.*

Supplementary Table S6. Distribution of potential effect modifiers across weekly-volume nodes

| **Potential effect modifier** | **45–90 min/week** | **91–150 min/week** | **151–360 min/week** |
| --- | --- | --- | --- |
| **Study/sample context** | | | |
| Studies, k | 10 | 10 | 4 |
| Completed follow-up participants, n (E+C) | 853 | 635 | 304 |
| **Baseline symptom burden** | | | |
| Baseline EPDS, median (range) | 13.22 (7.38–19.32) | 17.30 (8.45–20.18) | 7.52 (5.00–13.60) |
| EPDS-based eligibility threshold used, n (%) | 5 (50.0%) | 8 (80.0%) | 1 (25.0%) |
| Reported EPDS eligibility threshold(s) | ≥13; ≥12; >9; ≥10 | ≥12; >12; >9; ≥10; ≥13 | ≥10 |
| **Postpartum timing** | | | |
| Intervention start ≤6 weeks postpartum, n (%) | 6 (60.0%) | 3 (30.0%) | 0 (0.0%) |
| Intervention start >6 weeks postpartum, n (%) | 1 (10.0%) | 0 (0.0%) | 1 (25.0%) |
| Variable/broad start window or not reported, n (%) | 3 (30.0%) | 7 (70.0%) | 3 (75.0%) |
| **Intervention duration** | | | |
| Duration, weeks, median (range) | 8.0 (4–12) | 12.0 (4–24) | 10.0 (4–12) |
| Duration not reported, n | 0 | 1 | 0 |
| **Supervision** | | | |
| Supervised, n (%) | 6 (60.0%) | 7 (70.0%) | 3 (75.0%) |
| Unsupervised, n (%) | 4 (40.0%) | 3 (30.0%) | 1 (25.0%) |
| **Co-intervention** | | | |
| Exercise plus co-intervention, n (%) | 4 (40.0%) | 5 (50.0%) | 1 (25.0%) |
| Exercise-only, n (%) | 6 (60.0%) | 5 (50.0%) | 3 (75.0%) |
| **Comparator type** | | | |
| Active comparator, n (%) | 4 (40.0%) | 5 (50.0%) | 0 (0.0%) |
| Passive/usual-care comparator, n (%) | 6 (60.0%) | 5 (50.0%) | 4 (100.0%) |

*Note. Values are descriptive; no formal between-node hypothesis testing was performed. Baseline EPDS is the median (range) of study-level averages of the intervention- and comparator-arm baseline means. “≤6 weeks” and “>6 weeks” indicate studies whose reported start timing lay wholly within that category; broad/overlapping start windows or unreported timing were classified as variable/broad or not reported. Co-intervention refers to adjunctive non-exercise components in the active arm. Comparator type follows the finalized active versus passive/usual-care classification used in the comparator sensitivity analyses. Percentages use the number of studies within each node as the denominator.*

Supplementary Table S7. CINeMA-informed certainty of evidence for exercise-type network comparisons

| **Comparison** | **MD (95% CI)** | **95% prediction interval** | **Within-study bias** | **Reporting bias** | **Indirectness** | **Imprecision** | **Heterogeneity** | **Incoherence** | **Overall confidence** |
| --- | --- | --- | --- | --- | --- | --- | --- | --- | --- |
| Walking/cycling vs Aerobic exercise | -0.94 (-3.89, 2.01) | -6.77, 4.89 | Some concerns | Undetected | Some concerns | Some concerns | Some concerns | Major concerns* | Very low |
| Yoga/mind-body exercise vs Walking/cycling | -1.68 (-4.83, 1.47) | -7.62, 4.26 | Some concerns | Undetected | Some concerns | Some concerns | Some concerns | Major concerns* | Very low |
| Yoga/mind-body exercise vs Aerobic exercise | -2.62 (-5.16, -0.08) | -8.26, 3.02 | Some concerns | Undetected | Some concerns | No concerns | Some concerns | Major concerns* | Very low |
| Walking/cycling vs Control | -2.65 (-5.12, -0.19) | -8.25, 2.95 | Some concerns | Undetected | No concerns | No concerns | Some concerns | Major concerns* | Very low |
| Aerobic exercise vs Control | -1.71 (-3.32, -0.10) | -6.99, 3.57 | Some concerns | Undetected | No concerns | No concerns | Some concerns | Major concerns* | Very low |
| Yoga/mind-body exercise vs Control | -4.33 (-6.30, -2.37) | -9.73, 1.07 | Some concerns | Undetected | No concerns | No concerns | Some concerns | Major concerns* | Very low |

*Note. EPDS = Edinburgh Postnatal Depression Scale; CI = confidence interval. Certainty was assessed across within-study bias, reporting bias, indirectness, imprecision, heterogeneity, and incoherence using a CINeMA-informed approach. A 4-point EPDS difference was used as an evidence-informed clinically important threshold, defining an equivalence range of −4 to +4 points. Within-study bias was informed by the final RoB 2 judgments; reporting bias was rated as undetected based on the updated search, trial-registry searches, and absence of clear small-study effects, while acknowledging limited comparison-specific power. Indirectness judgments were informed by the distribution of effect modifiers in Supplementary Table S5. Imprecision and heterogeneity were evaluated against the defined equivalence range using 95% CIs and prediction intervals. *Neither network contained closed loops; therefore incoherence could not be empirically evaluated. “Major concerns” reflects this non-assessability rather than detected incoherence. Overall confidence was judged across domains while avoiding duplicate downgrading for related concerns.*

*S*upplementary Table S8. CINeMA-informed certainty of evidence for weekly-volume network comparisons

| **Comparison** | **MD (95% CI)** | **95% prediction interval** | **Within-study bias** | **Reporting bias** | **Indirectness** | **Imprecision** | **Heterogeneity** | **Incoherence** | **Overall confidence** |
| --- | --- | --- | --- | --- | --- | --- | --- | --- | --- |
| 45–90 vs 91–150 min/week | -0.52 (-3.10, 2.07) | -6.48, 5.44 | Some concerns | Undetected | Major concerns | Some concerns | Some concerns | Major concerns* | Very low |
| 45–90 vs 151–360 min/week | -2.04 (-5.40, 1.33) | -8.38, 4.30 | Some concerns | Undetected | Major concerns | Some concerns | Some concerns | Major concerns* | Very low |
| 91–150 vs 151–360 min/week | -1.52 (-4.93, 1.89) | -7.89, 4.85 | Some concerns | Undetected | Major concerns | Some concerns | Some concerns | Major concerns* | Very low |
| 45–90 min/week vs Control | -3.30 (-5.09, -1.52) | -8.96, 2.36 | Some concerns | Undetected | No concerns | No concerns | Some concerns | Major concerns* | Very low |
| 91–150 min/week vs Control | -2.79 (-4.65, -0.92) | -8.48, 2.90 | Some concerns | Undetected | No concerns | No concerns | Some concerns | Major concerns* | Very low |
| 151–360 min/week vs Control | -1.27 (-4.12, 1.59) | -7.36, 4.82 | Some concerns | Undetected | No concerns | Some concerns | Some concerns | Major concerns* | Very low |

*Note. EPDS = Edinburgh Postnatal Depression Scale; CI = confidence interval. Certainty was assessed across within-study bias, reporting bias, indirectness, imprecision, heterogeneity, and incoherence using a CINeMA-informed approach. A 4-point EPDS difference was used as an evidence-informed clinically important threshold, defining an equivalence range of −4 to +4 points. Within-study bias was informed by the final RoB 2 judgments; reporting bias was rated as undetected based on the updated search, trial-registry searches, and absence of clear small-study effects, while acknowledging limited comparison-specific power. Indirectness judgments were informed by the distribution of effect modifiers in Supplementary Table S6, particularly baseline EPDS, EPDS-based eligibility, postpartum timing, and comparator type. Imprecision and heterogeneity were evaluated against the defined equivalence range using 95% CIs and prediction intervals. *Neither network contained closed loops; therefore incoherence could not be empirically evaluated. “Major concerns” reflects this non-assessability rather than detected incoherence. Overall confidence was judged across domains while avoiding duplicate downgrading for related concerns.*
